# Unlocking the Potential of Ti_3_C_2_T_x_ MXene: Present Trends and Future Developments of Gas Sensing

**DOI:** 10.3390/mi16020159

**Published:** 2025-01-29

**Authors:** Aviraj M. Teli, Sagar M. Mane, Rajneesh Kumar Mishra, Wookhee Jeon, Jae Cheol Shin

**Affiliations:** 1 Division of Electronics and Electrical Engineering, Dongguk University-Seoul, Seoul 04620, Republic of Korea; avteli.teli@gmail.com; 2 Department of Fiber System Engineering, Yeungnam University, 280 Dehak-Ro, Gyeongsan 38541, Gyeongbuk, Republic of Korea; manesagar99@gmail.com; 3 Department of Physics, Yeungnam University, Gyeongsan 38541, Gyeongbuk, Republic of Korea; 4 Department of Semiconductor, Convergence Engineering, Sungkyunkwan University, Suwon 16419, Gyeonggi, Republic of Korea; wookie92@skku.edu

**Keywords:** Ti_3_C_2_T_x_ MXenes, surface functionalization, properties, sensor technology, gas sensing, future prospects

## Abstract

In recent years, the need for future developments in sensor technology has arisen out of the changing landscape, such as pollution monitoring, industrial safety, and healthcare. MXenes, a 2D class of transition metal carbides, nitrides, and carbonitrides, have emerged as a particularly promising group in part due to their exceptionally high conductivity, large area, and tunable surface chemistry. Proposed future research directions, including material modification and novel sensor designs, are presented to maximize Ti_3_C_2_T_x_ MXene-based sensors for various gas sensing applications. While recent progress in Ti_3_C_2_T_x_ MXene-based gas sensors is reviewed, we consolidate their material properties, fabrication strategy, and sensing mechanisms. Further, the significant progress on the synthesis and applications of Ti_3_C_2_T_x_ MXene-based gas sensors, as well as the innovative technologies developed, will be discussed in detail. Interestingly, the high sensitivity, selectivity, and quick response times identified in recent studies are discussed, with specificity and composite formation highlighted to have a significant influence on sensor performance. In addition, this review highlights the limitations witnessed in real-life implementability, including stability, the possibility of achieving reproducible results, and interaction with currently available technologies. Prospects for further work are considered, emphasizing increased production scale, new techniques for synthesis, and new application areas for Ti_3_C_2_T_x_ MXenes, including electronic nose and environmental sensing. Contemplating the existing works, further directions and the development framework for Ti_3_C_2_T_x_ MXene-based gas sensors are discussed.

## 1. Introduction

Recently, there has been a great deal of interest in the development of advanced and versatile gas sensors as they are important for environmental monitoring, industrial safety, and healthcare. Volatile organic compounds (VOCs) are a major source of air pollution, and of concern for human health and the environment [1]. Air quality and plans for local pollution control means are dependent on monitoring the VOC level. Commonly used industry applications of VOCs are chemical production, painting, and surface coating [2]. These sectors are monitored to ensure compliance with workplace safety and regulatory spaces to ensure that these sectors are thriving. A representative VOC, acetone, has medical and industrial uses [3]. The medical association of elevated acetone levels in human exhalation has already been documented, and the condition is used as a diagnostic and management tool [4]. Industrially, acetone is used as a solvent, so its presence in the workplace must be monitored to avoid exposure hazards and promote workplace safety [5]. In addition, agricultural and industrial wastewater are polluted by ammonia [6]. Monitoring ammonia is vital to guard against water contamination and to determine the adequacy of water treatment processes [7]. Ammonia is also widely used in refrigeration systems and the fertilizer industry [8]. The risk to workers’ health when releasing ammonia is critical to occupational safety and must be monitored [9]. On the other hand, industrial processes, including vehicle exhaust, are the source of most of the NO_2_ emitted [10]. Also, levels of NO_2_ are used for air quality monitoring and the control of pollution [11]. In addition, NO_2_ concentration measurements are also essential to safeguard public health and serve as input into regulatory policy to reduce air pollution [12]. Excessive exposure to NO_2_ is associated with chronic respiratory problems and the worsening of asthma [13]. The ability of advanced gas sensors to address the monitoring needs of these gases is critically vital for protecting human health, achieving industrial safety, and mitigating environmental impacts.

Two-dimensional (2D) materials, particularly MXenes, show great promise due to their exceptional structural, electrical, and chemical properties and offerings among the emerging solutions [14]. The general formula for MXenes, which represents the selective etching of precursor MAX phases, has been defined as M_n+1_X_n_T_x_, where M stands for a transition metal, such as titanium (Ti), scandium (Sc), niobium (Nb), molybdenum (Mo), tantalum (Ta), or vanadium (V), which contributes to metallic conductivity and machinability; X denotes carbon or nitrogen, forming strong covalent bonds with the transition metal to impart hardness and resistance to oxidation and corrosion; and T_x_ pertains to the termination of the surface, such as -OH, -O, or -F [15,16,17,18,19]. Specifically, Ti_3_C_2_T_x_ MXene, a member of the MXene family derived from layered transition metal carbides or nitrides, has demonstrated exceptional potential for gas sensing applications [20]. Ti_3_C_2_T_x_ MXenes exhibit remarkable electronic properties attributed to their two-dimensional (2D) structure, comprising transition metal carbide layers interleaved with functional groups such as hydroxyl and fluorine terminations [21]. Moreover, the synthesis of Ti_3_C_2_T_x_ MXene is a rapidly evolving field with several methods available, each offering unique advantages and challenges. The most common method for synthesizing Ti_3_C_2_T_x_ MXene is through hydrofluoric acid (HF) etching [22,23]. In this approach, the MAX phase is immersed in concentrated HF, which selectively reacts with aluminum, forming AlF_3_ and liberating hydrogen gas. The resultant Ti_3_C_2_T_x_ MXene contains functional groups such as -F, -OH, and -O, enhancing its hydrophilicity. While this method is effective and straightforward, the use of highly toxic HF poses significant safety concerns, necessitating careful handling and disposal. Therefore, to address the hazards of HF, an alternative method involves in situ HF generation by reacting fluoride salts, such as LiF or NaF, with hydrochloric acid (HCl) [24,25]. This generates HF in a controlled manner, which then etches the Al layer from the MAX phase. On the other hand, Ti_3_C_2_T_x_ MXene is also synthesized using alkali treatment, which employs strong bases like sodium hydroxide (NaOH) to etch the MAX phase [26]. This fluoride-free approach reduces environmental and safety concerns, but it is less effective and commonly used than acid-based methods. A recent innovation is molten salt etching, where the MAX phase is treated with molten salts, such as ZnCl_2_ or LiCl-KCl, at high temperatures (550–700 °C) [27]. This method not only removes the aluminum layer but also allows for the tuning of the surface chemistry of the resulting MXenes. However, it requires specialized equipment and a higher energy input. These methods will enable the production of high-quality MXenes tailored for various applications. The structure of Ti_3_C_2_T_x_ MXene enables high electronic conductivity and offers plentiful active sites for gas molecule adsorption, thereby improving gas sensing performance [28]. Moreover, the surface chemistry of Ti_3_C_2_T_x_ MXene can be tailored through selective functionalization or compositional modifications, offering versatility in the tuning of sensor selectivity and sensitivity towards specific gases [29]. Ti_3_C_2_T_x_ MXene-based gas sensors have witnessed significant advancements in recent years, marked by efforts to enhance sensor performance metrics, including response time, selectivity, and detection limits [30]. These efforts are complemented by computational studies and theoretical models that provide insights into the underlying gas sensing mechanisms at the atomic and molecular levels. Furthermore, integration strategies with novel nanomaterials, advanced fabrication techniques, and innovative sensor configurations have further propelled the development of Ti_3_C_2_T_x_ MXene-based gas sensors toward practical applications [31,32,33]. Gas sensors are indispensable for industrial safety, environmental monitoring, and healthcare diagnostics. Despite wide use, conventional metal oxide-based gas sensors are limited to high operating temperatures and insufficient sensitivity at low concentrations. Interestingly, room-temperature gas sensing applications of MXenes, with their metallic conductivity, tunable surface chemistry, and hydrophilicity, are proposed to be a breakthrough for the material.

This review aims to provide a comprehensive understanding of the structure, morphology, and recent developments in the gas sensing applications of Ti_3_C_2_T_x_ MXene. It discusses the fundamental electronic and optical properties of Ti_3_C_2_T_x_ MXene that contribute to its effectiveness as a gas sensor, highlights vital achievements, and outlines current challenges and future directions for harnessing its full potential in gas sensing applications. In addition, this review provides a roadmap for researchers and engineers seeking to exploit Ti_3_C_2_T_x_ MXene-based gas sensors for diverse and demanding environmental and industrial monitoring scenarios by synthesizing and analyzing the latest research findings. Finally, the review offers a summary and a discussion on outlooks for future progress.

## 2. Structural, Morphological, and Elemental Analyses

**Figure 1:**
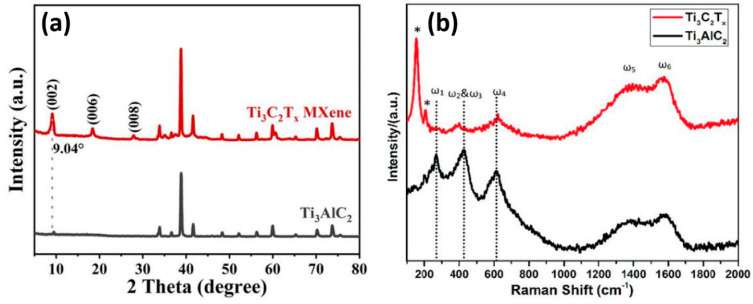
(**a**) XRD patterns of Ti_3_AlC_2_ and Ti_3_C_2_T_x_ MXenes. Reproduced with permission from Ref. [37]. Copyright (2021) Springer Nature. (**b**) Raman spectra of Ti_3_AlC_2_ and Ti_3_C_2_T_x_ MXenes (* represents out of plane vibrations of Ti and C). Reproduced with permission from Ref. [47]. Copyright (2021) MDPI.

X-ray diffraction (XRD) is a versatile method for determining the crystallographic nature of Ti_3_C_2_T_x_ MXene. Ti_3_C_2_T_x_ MXene usually exhibits a layered structure with characteristics resembling graphene oxide, which forms layers of transition metal atoms in between layers of carbon atoms [34]. Depending on the stacked layer thickness, the lattice plane (002) may be detected at 0.9–1.0 nm, which represents the interlayer distance of different Ti_3_C_2_T_x_ MXene layers [35]. Moreover, the reduced interlayer d-spacing in the range of 0.28 nm can be shown for the lattice plane (004), which exists at a smaller scattering angle compared to the (002) lattice plane of Ti_3_C_2_T_x_ MXene [36]. Yao et al. discussed the XRD spectra of Ti_3_AlC_2_ and Ti_3_C_2_T_x_ MXenes, as elucidated in Figure 1a [37]. It was observed that Ti_3_C_2_T_x_ MXene showed various lattice planes, such as (002), (004), and (008). Based on Raman analysis, the Raman spectrum of Ti_3_C_2_T_x_ exhibits peaks associated with a material structure, including disorder (D band), in-plane vibrations (G band), out-of-plane vibrations (M band), and signals from surface functional groups [38]. Such analysis provides valuable information on critical aspects such as the quality of the produced Ti_3_C_2_T_x_ MXenes, the defects present, and the surface chemistry [39]. The Raman D band of Ti_3_C_2_T_x_ concerns the vibrational modes related to disorder or defects in the MXene structure and is usually observed at an 800–1000 cm^−1^ wave number [40]. The Raman D band of Ti_3_C_2_T_x_ MXene is reactive to defects, including vacancies, surface-terminating groups like -OH or -F, or a distorted lattice [41]. The Raman G band of Ti_3_C_2_T_x_ MXene is usually observed at 1500–1600 cm^−1^ in the Raman spectrum [42]. In addition, the Raman G band is connected with the E_2g_ phonon mode of Ti_3_C_2_T_x_ MXene’s structure [43]. This mode also entails oscillations of the carbon atoms in the Ti_3_C_2_ plane. It was reported that the intensity and position of the Raman G band can provide information about the structure and bonding of Ti_3_C_2_T_x_ MXene. It was also observed that the Raman M band is related to the out-of-plane vibrations of the Ti atoms in the Ti_3_C_2_T_x_ structure [44]. Typically found in the range of 300 to 400 cm^−1^, the intensity of its signal can depend on stacking and interlayer interactions between the Ti_3_C_2_T_x_ MXene sheets [45]. Besides the known strong D, G, and M Raman bands, Ti_3_C_2_T_x_ Raman spectra can also feature additional bands associated with the specific vibrational modes of functional groups covalently attached to the Ti_3_C_2_T_x_ MXene surface (T_x_ groups) [46]. Iqbal et al. discussed the Raman spectra of Ti_3_AlC_2_ and Ti_3_C_2_T_x_ MXenes, as depicted in Figure 1b [47]. The vibrational Raman peaks ω_1_, ω_2_, ω_3_, and ω_4_ are characteristic of the shear and longitudinal oscillations of Ti and Al atoms, which are observed in the spectra of the MAX phase at approximately 269 cm^−1^, 423 cm^−1^, and 613 cm^−1^. Furthermore, the ω_1_ peak due to Al vibrations disappears in the Mxene spectrum, indicating the successful etching of Al atoms and incorporation into the Mxene structure. The Mxene spectrum exhibits a prominent peak at about 151 cm^−1^ that suggests this Ti_3_C_2_ is oxidized, presumably as a result of increased surface reactivity. Additionally, a peak at 208 cm^−1^, due to out-of-plane vibrations in the Ti and C, indicates the incorporation of functional groups such as -OH and -F during the etching process.

**Figure 2:**
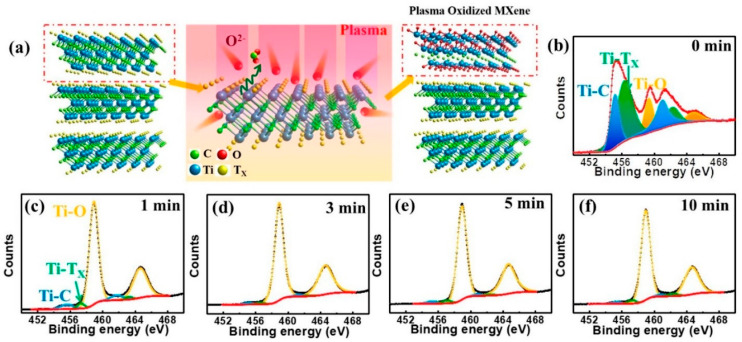
(**a**) A schematic illustration of the Ti_3_C_2_T_x_ MXene structure before and after oxygen plasma treatment, and (**b**–**f**) the Ti 2p core-level XPS spectra of the MXene exposed to oxygen plasma for 0, 1, 3, 5, and 10 min, respectively. Reproduced with permission from Ref. [55]. Copyright (2021) American Chemical Society.

The nature of the functional groups, such as -OH and -F, and their interaction with the Ti_3_C_2_ lattice leads to the appearance of these Raman bands at different frequencies [48]. Interestingly, XPS is a valuable tool for characterizing the elemental composition, chemical bonding states, and surface chemistry of Ti_3_C_2_T_x_. The Ti 2p core level in XPS of Ti_3_C_2_T_x_ is found at about 458–460 eV for Ti^2+^ and 454–456 eV for Ti^3+^ [49]. Additionally, the Ti^2+^ peak indicates titanium in a reduced state, generally related to Ti-C bonding [50]. Also, the Ti^3+^ peak represents the higher binding-energy states of the titanium in its oxidized form, commonly linked with Ti-O or Ti-F bonding [51]. The binding energies may depend slightly on surface termination (T_x_) factors. The C 1s peak is typically observed at 281–284 eV with the ability to deconvolute to C-C/C-Ti, C-OH, and C=O [52] in the Ti_3_C_2_ carbon layers, or directly bound to titanium. The surface binds with the hydroxyl groups (C-OH) of carbon atoms on the surface. Oxygen on the MXene surface is indicated by the presence of carbonyl (C=O) groups [53]. This surface chemistry and functionalization of Ti_3_C_2_T_x_ is revealed in the relative intensities and positions of these peaks. Interestingly, the O 1s peaks appear at 530–532 eV, and F 1s peaks at 685–688 eV [54]. In addition, the XPS can distinguish further surface functional groups from hydroxyl (-OH), oxide (-O), and fluoride (-F) terminations. Understanding the surface reactivity, electronic properties, and interactions of the Ti_3_C_2_T_x_ is dependent upon the presence and relative abundance of these groups. Wang et al. reported the plasma-oxidized Ti_3_C_2_T_x_ MXene and its XPS spectra to discuss its chemical composition, as shown in Figure 2a–f [55]. Figure 2a shows the changes induced at the atomic level, which are presented by the schematic representation of the Ti_3_C_2_T_x_ MXene structure before and after oxygen plasma treatment. The MXene structure before plasma treatment consists of layers of titanium carbide (Ti_3_C_2_) with surface terminations -OH, -F, and -O, which result from the etching process in the synthesis. It is made clear that these surface terminations are essential to Ti_3_C_2_T_x_ MXene’s electrical, chemical, and mechanical properties. In the oxygen plasma-treated Ti_3_C_2_T_x_ MXene, the existing terminations are removed or altered, and oxygen functionalities are introduced.

Figure 2b illustrates the XPS spectra of the Ti 2p core-level XPS spectra, used for a detailed study of the oxygen plasma-treated surface chemistry. Figure 2b shows that the XPS spectrum of Ti_3_C_2_T_x_ MXene recorded at 0 min features prominent peaks due to Ti-T_x_, Ti-C, and Ti-O bonds. On the other hand, the XPS spectra in Figure 2c–f display progressive shifts and changes in the Ti_2p_ peak intensity with increasing plasma exposure time (1, 3, 5, and 10 min). These transformations correspond to the switching of surface terminations from oxygen-deficient to oxygen-rich groups. Therefore, these unique structural and elemental characterization techniques are pivotal in determining the structural, electronic, surface termination, and chemical properties of Ti_3_C_2_T_x_ MXene. These features make Ti_3_C_2_T_x_ MXenes highly suitable for applications in gas sensing, where sensitivity, selectivity, and stability are critical requirements. In addition, titanium (Ti) contributes to the high electrical conductivity of Ti_3_C_2_T_x_ MXenes, facilitating the fast charge transport that is essential for improved gas sensing applications. Additionally, Ti forms strong covalent bonds with carbon, ensuring structural stability under various conditions, which is also vital for the gas sensor’s stability. The d-orbital electrons of Ti also enhance gas detection, enabling stronger interactions with gas molecules, which is vital for achieving high sensitivity in gas sensors. The carbon layers within the Ti_3_C_2_T_x_ MXene structure provide a conductive backbone and mechanical flexibility, which are advantageous for flexible and wearable gas sensing devices. Surface terminations (-T_x_), such as -O, -OH, and -F, introduced during the synthesis process significantly influence the chemical reactivity, hydrophilicity, and surface energy of Ti_3_C_2_T_x_ MXene. These functional groups enhance the adsorption of polar gas molecules, such as NH_3_ or NO_2_, by creating active sites for interaction. The layered structure of Ti_3_C_2_T_x_ MXenes, with tunable interlayer spacing, further supports gas diffusion and adsorption, improving their selectivity for specific gases. This ability to tailor the surface chemistry and structure is a key advantage of MXenes over other materials used for gas sensing.

**Figure 3:**
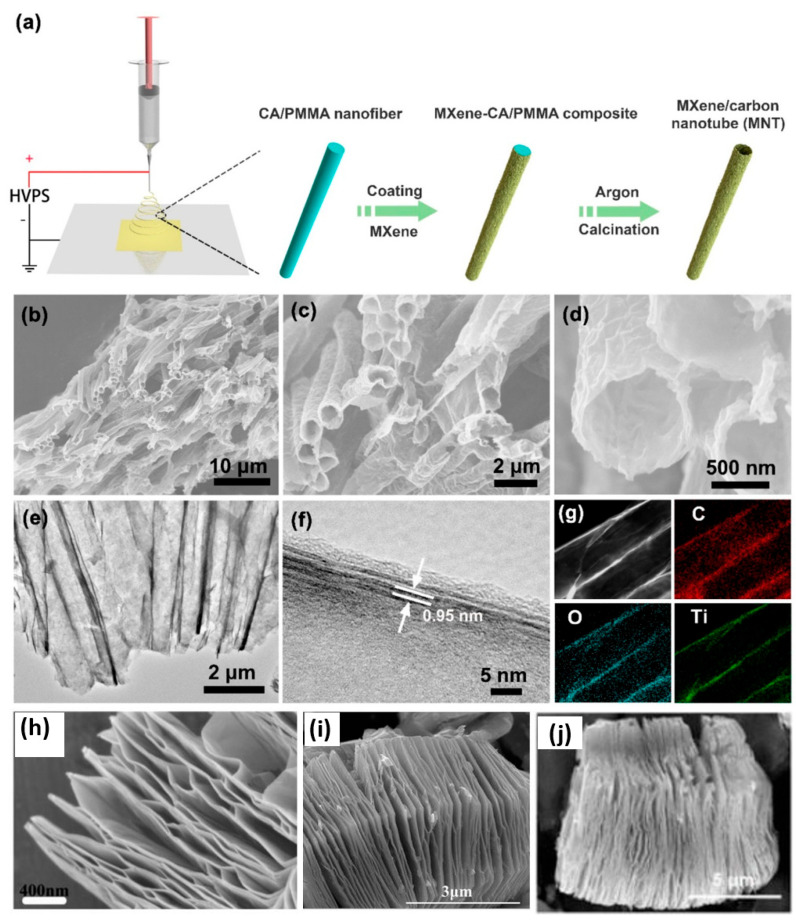
(**a**) A schematic illustration of the synthetic route for the Ti_3_C_2_T_x_ MXene/carbon nanotube; (**b**–**d**) SEM images of the morphology of the Ti_3_C_2_T_x_ MXene/carbon nanotube at various magnifications; (**e**,**f**) high-resolution transmission electron microscopy (HRTEM) images of the internal structure and lattice fringes of the Ti_3_C_2_T_x_ MXene/carbon nanotube; and (**g**) elemental mapping of C, O, and Ti elements of the Ti_3_C_2_T_x_ MXene/carbon nanotube showing the uniformity of the material composition. Reproduced with permission from Ref. [56]. Copyright (2021) American Chemical Society. (**h**) A high-magnification SEM image of MXene–Ti_3_C_2_. Reproduced with permission from Ref. [57]. Copyright (2015) Institute of Physics. (**i**) An SEM image of Ti_3_C_2_T_x_ MXene. Reproduced with permission from Ref. [58]. Copyright (2020) Wiley-VCH GmbH. (**j**) An SEM image of Ti_3_C_2_T_x_ MXene. Reproduced with permission from Ref. [59]. Copyright (2024) American Chemical Society.

Hu et al. reported on hierarchical Ti_3_C_2_T_x_ MXene/carbon nanotubes as illustrated in Figure 3a–f [56]. Figure 3a–g elucidate the schematic synthesis process, morphology, structure, and elemental characteristics of the hierarchical Ti_3_C_2_T_x_ MXene/carbon nanotubes, reflecting its architecture and composition. In the schematic [Figure 3a], the Ti_3_C_2_T_x_ MXene/carbon nanotubes are prepared, deposited onto composite nanofibers, and processed to form a tubular structure via electrospinning. By using self-sacrificial templating, the formation of the rigid, aligned tubular architecture of Ti_3_C_2_T_x_ MXene/carbon nanotubes is facilitated. Figure 3b–d reveal that the morphology of the Ti_3_C_2_T_x_ MXene/carbon nanotubes is highly organized, consisting of a tubular form as determined by SEM. In addition, the calcined nanotubes have an average diameter of approximately 1.5 µm, exhibiting a wrinkled surface. An optimal MXene to polymer ratio during synthesis was found to preserve the tubular morphology. Further insights into the exact structure of the Ti_3_C_2_T_x_ MXene/carbon nanotubes are provided by high-resolution transmission electron microscopy (HRTEM) images, as shown in Figure 3e,f, which show the ultrathin MXene layers of the Ti_3_C_2_T_x_ MXene/carbon nanotubes. The layered structure turns out to be ductile and folded, with interlayer spacings expanded to approximately 0.95 nm. In addition, the inner surface of the nanotubes is covered by a thin layer of carbon obtained by the pyrolysis of the polymer template, which contributes to the stability of the structure. Figure 3g shows the elemental mapping of the Ti_3_C_2_T_x_ MXene/carbon nanotubes and confirms the uniform C, O, and Ti distribution throughout the Ti_3_C_2_T_x_ MXene/carbon nanotube heterostructure. The successful integration of Ti_3_C_2_T_x_ MXene with carbon nanotubes, confirmed by this even dispersion, also supports the stability of their structure. In addition, Wang et al. discussed SEM imagery of MXene–Ti_3_C_2_, which features a layered structure, as depicted in Figure 3h [57]. The high-magnification SEM image in Figure 3h highlights the distinct multilayer structure of the MXene–Ti_3_C_2_, with an average layer thickness of less than 20 nm. This well-exfoliated 2D layered structure plays a critical role in enhancing the gas sensing properties of the MXene–Ti_3_C_2_. The multilayered configuration of MXene–Ti_3_C_2_ provides a significantly high surface area, allowing a greater density of adsorption sites for gas molecules. This increased surface area improves the sensitivity of the MXene–Ti_3_C_2_ by enabling more interactions with target gases. Wang et al. reported on SEM imagery of Ti_3_C_2_T_x_ MXene, as portrayed in Figure 3i, revealing its characteristic accordion-like morphology with significant interlayer spacing [58]. This unique morphology is highly advantageous for gas sensing applications due to several key factors, such as the accordion-like structure providing a substantially increased surface area, ensuring more active sites on the surface of the Ti_3_C_2_T_x_ MXene, which is critical for the adsorption and desorption of various gas molecules. Zhang et al. described SEM imagery of Ti_3_C_2_T_x_ MXene, as exposed in Figure 3j, demonstrating the transformation of Ti_3_AlC_2_ MAX into Ti_3_C_2_T_x_ MXene following the etching of the Al layer [59]. This process results in a 2D layered morphology, which significantly enhances the properties of the Ti_3_C_2_T_x_ MXene, making it highly suitable for gas sensing applications. The removal of the Al layer creates expanded interlayer spacing and a high surface area, which is crucial for improving gas adsorption capacity. In addition, the metallic conductivity of Ti_3_C_2_T_x_ MXene is another vital characteristic for gas sensing applications. Ti_3_C_2_T_x_ MXenes are ideal for detecting changes in electrical resistance or conductivity caused by gas adsorption. When target gas molecules interact with the surface of Ti_3_C_2_T_x_ MXenes, the electrical changes are measurable, allowing for efficient gas detection, ensuring a rapid response to the presence of gas molecules, which is essential for real-time sensing applications. Moreover, modifying the surface functional groups of Ti_3_C_2_T_x_ MXene, such as -OH, -F, or -O are essential to improve the selective detection for specific gas molecules, which are filled with various interfering gases. Such functionalization ensures that the Ti_3_C_2_T_x_ MXene responds predominantly to the desired gas, reducing interference from other gases in the environment.

## 3. Properties

### 3.1. Optical

Ti_3_C_2_T_x_ MXene exhibits interesting optical properties due to its unique structure and composition. Ti_3_C_2_T_x_ MXene is generally transparent in the visible to near-infrared spectrum, which makes it suitable for applications in transparent electrodes and optoelectronics [60]. This transparency is attributed to its layered structure, where the Ti_3_C_2_T_x_ MXene layers allow light to pass through. Depending on their specific composition and structure, Ti_3_C_2_T_x_ MXenes can exhibit plasmonic resonances in the infrared and terahertz frequencies [61]. These plasmonic properties are useful in sensing applications and for enhancing light–matter interactions. Ti_3_C_2_T_x_ MXene shows absorption in the ultraviolet (UV) region, primarily due to transitions between the valence and conduction bands [62]. The absorption edge typically covers into the visible spectrum, influencing its optical properties [63]. Ti_3_C_2_T_x_ MXene is reported to have a direct band gap, which means that the energy difference between the valence band and the conduction band occurs at the same momentum (k) point in the Brillouin zone [64]. Direct band gaps favor optoelectronic applications such as photodetectors and light-emitting devices. The band gap of Ti_3_C_2_T_x_ MXene can be influenced by various factors, including surface functionalization, which alters the optical properties. By adjusting the surface terminations (T_x_), researchers can tune the band gap of Ti_3_C_2_T_x_ MXene for specific applications [65]. The experimental studies have reported band gaps in Ti_3_C_2_T_x_ MXenes ranging from approximately 0.5 eV to 2.5 eV, depending on the specific synthesis method, degree of surface functionalization, and layer thickness [66]. Modulating the band gap makes Ti_3_C_2_T_x_ MXene versatile for various applications, from energy storage to photovoltaics [67]. Furthermore, Ti_3_C_2_T_x_ MXene exhibits transparency in the visible spectrum, plasmonic resonances in the infrared, and a tunable direct band gap that can be tailored through surface modifications [68]. These properties make Ti_3_C_2_T_x_ MXene a promising material for next-generation optoelectronics, sensors, and other emerging technologies. Further, the photoluminescence (PL) peaks of Ti_3_C_2_T_x_ MXene arise primarily from surface functional groups and defects [69]. These groups can introduce energy levels within the band gap of the Ti_3_C_2_T_x_ MXene, leading to the emission of light when excited [70]. The PL emission spectrum of Ti_3_C_2_T_x_ MXene typically depends on the excitation wavelength. This dependence suggests that different energy states or defects of the Ti_3_C_2_T_x_ MXene contribute to this emission, each responding differently to excitation energy [71]. The emission spectrum of Ti_3_C_2_T_x_ MXene ranges from visible to near-infrared wavelengths [72]. The emitted light can be tuned by modifying the surface chemistry through functionalization or controlling the synthesis conditions of the Ti_3_C_2_T_x_ MXene [73]. The PL properties make Ti_3_C_2_T_x_ MXene suitable for sensing applications, such as detecting gases, biomolecules, or environmental pollutants. Ti_3_C_2_T_x_ MXene could potentially be integrated into optoelectronic devices, which emit light under certain conditions, opening possibilities for applications in LEDs, displays, and even as components in photonic circuits [74]. The near-infrared emissions of Ti_3_C_2_T_x_ MXene are particularly advantageous for biomedical imaging due to their deeper tissue penetration and reduced autofluorescence from biological samples [75]. The FTIR spectra of Ti_3_C_2_T_x_ MXene typically show characteristic peaks related to oxygen-containing functional groups (OH, C=O), carbonaceous species (C-H, C-C), Ti-C bonds, and interlayer water [76]. These peaks’ exact positions and intensities provide information about the synthesis method, surface chemistry, and oxidation state of the Ti_3_C_2_T_x_ MXene. The hydroxyl groups (OH) are typically observed around 3000–3700 cm^−1^ (broad peak) and around 1400–1600 cm^−1^ (shoulder peak) [77]. The presence of hydroxyl groups in the Ti_3_C_2_T_x_ MXene indicates the presence of water or surface hydroxyls. On the other hand, the carbonyl groups (C=O) appear around 1700–1750 cm^−1^, which indicates the presence of surface-adsorbed species or oxidation states on the Ti_3_C_2_T_x_ MXene. Further, oxide groups (Ti-O-Ti) in the Ti_3_C_2_T_x_ MXene are observed around 400–800 cm^−1^ [78]. These bands indicate the presence of TiO_x_ species in the Ti_3_C_2_T_x_ MXene, which can form due to oxidation during synthesis or exposure to air. In addition, the C-H stretching and bending of Ti_3_C_2_T_x_ MXene can be observed around 2800–3000 cm^−1^ and 1300–1500 cm^−1^, respectively [79]. Also, C-C and C=C bonds in Ti_3_C_2_T_x_ MXene typically appear in the 1000–1500 cm^−1^ range [80]. The Ti-C bonds of Ti_3_C_2_T_x_ MXene are a broad feature in the 600–800 cm^−1^ region [81]. The exact position can vary depending on the titanium’s chemical environment and oxidation state. Additional peaks related to surface functional groups such as -F, -Cl, or -O may be observed depending on the synthesis method and subsequent processing [82].

**Figure 4:**
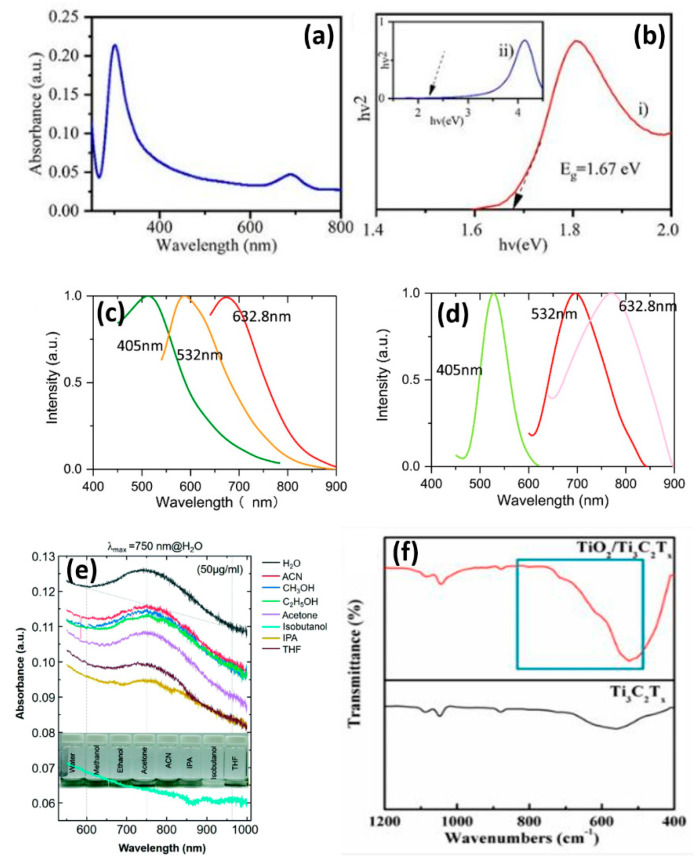
(**a**) UV–visible absorption spectrum and (**b**) related optical band gap of Ti_3_C_2_T_x_ MXene. Reproduced with permission from Ref. [83]. Copyright (2024) Springer Nature. PL spectrum of Ti_3_C_2_T_x_ MXene (**c**) without surface modification and (**d**) with surface modification at various excitation wavelengths. Reproduced with permission from Ref. [84]. Copyright (2019) Elsevier. (**e**) UV–visible NIR spectra of Ti_3_C_2_T_x_ MXene and optical image in different solvents. Reproduced with permission from Ref. [85]. Copyright (2020) Royal Society of Chemistry. (**f**) FTIR spectra of TiO_2_/Ti_3_C_2_T_x_ and Ti_3_C_2_T_x_ MXenes. Reproduced with permission from Ref. [86]. Copyright (2019) MDPI.

Kumar et al. discussed the absorption spectrum and evaluated the optical band gap of Ti_3_C_2_T_x_ MXene, as depicted in Figure 4a,b [83]. Figure 4a reveals the UV–visible absorption spectrum of Ti_3_C_2_T_x_ MXene, which illustrates a sharp peak at 300 nm attributed to surface functional groups (OH, O, F) created through the etching of the Al from the Ti_3_AlC_2_ MAX phase. In addition, a broad absorption peak centered at 690 nm is evidence of longitudinal plasmon resonance, typical of layered Ti_3_C_2_T_x_ MXene nanomaterials. Based on these features, it is clear that the material is highly absorbent of light over a wide spectral range. Moreover, Figure 4b discloses a band gap of approximately 1.67 eV for the Ti_3_C_2_T_x_ MXene, determined using Tauc’s plot, showing the semiconducting nature of Ti_3_C_2_T_x_ MXene. Zhang et al. discussed the photoluminescence (PL) spectra for excitation wavelength-dependent emission without the surface modification and with the surface modification of the Ti_3_C_2_T_x_ MXene, as portrayed in Figure 4c,d [84]. Figure 4c depicts the PL spectrum of the Ti_3_C_2_T_x_ MXene without surface modification. It illustrates three strong emissions from the Ti_3_C_2_T_x_ MXene at 503 nm (green) for 405 nm excitation, 597 nm (yellow) for 532 nm excitation, and 652 nm (red) for 632.8 nm excitation. Further, the redshift of the PL spectrum with increasing excitation wavelength indicates that the PL arises from defect states, with Ti_3_C_2_T_x_ MXene being deficient of the band gap that would be required for such emissions, and is likely associated with interspersed TiO_2_ clusters. On the other hand, Figure 4d portrays that the PL peaks of the surface-modified Ti_3_C_2_T_x_ MXene are further redshifted to 530 nm (green), 701 nm (yellow), and 759 nm (red). In the modification, the density and diversity of the defect energy states may be increased, broadening the range of PL emissions and decreasing the PL energy by 0.1–0.3 eV. These results emphasize the importance of defect engineering for tuning the optical properties of Ti_3_C_2_T_x_ MXene. Elumalai et al. discussed the UV–visible NIR spectra of Ti_3_C_2_T_x_ MXene, as described in Figure 4e [85]. Broad plasmon peaks originating from surface plasmon resonances (SP) and interband transitions inherent to Ti_3_C_2_T_x_ MXene’s structure are observed. Interestingly, the absorption characteristics are unchanged across different solvents, depicted in the spectra and inset photographs of Figure 4e. On the other hand, the robustness of the optical behavior of Ti_3_C_2_T_x_ MXene is due to functional groups from the MXene surface (-F, -OH, -O) that control its interaction with light. Captivatingly, it possesses the adaptability to enhance its application in biosensing, chemical detection, and optoelectronic devices through the ability to fine-tune these properties by changing the solvent. Du et al. discussed the FTIR spectra of Ti_3_C_2_T_x_ MXene, as designated in Figure 4f [86]. Figure 4f unveils the Fourier Transform Infrared (FTIR) spectra showing the structural and chemical composition of TiO_2_/Ti_3_C_2_T_x_ and Ti_3_C_2_T_x_ MXenes. A characteristic peak of the anatase TiO_2_ phase appears, corresponding to well-known peaks within the range of 800–500 cm^−1^. In particular, the peak at 570 cm^−1^ is attributed to the deformation vibration of the Ti-O bond. The FTIR spectra display chemical modifications due to the interactions between the TiO_2_ nanoparticles and the Ti_3_C_2_T_x_ substrate, leading to a robust hybrid structure.

### 3.2. Magnetic

The titanium and carbon atoms present in Ti_3_C_2_T_x_ MXene typically render paramagnetic behavior. In addition, the magnetic properties of Ti_3_C_2_T_x_ MXene are very sensitive to surface terminations [87]. Typically surface functional groups govern the surface paramagnetic nature in Ti_3_C_2_T_x_ MXene, which in turn affects the electronic structure and magnetic moment of Ti atoms [88]. Additionally, it has been observed that the magnetic behavior of Ti_3_C_2_T_x_ MXene varies between different surface terminations [89]. Interestingly, the -OH terminations of Ti_3_C_2_T_x_ MXene are expected to increase the magnetic moment compared with -F or -O terminations because of the electron donation from -OH groups to the Ti atoms resulting in enhanced spin density [90]. Furthermore, density functional theory (DFT) calculations have been carried out to predict the magnetic properties of Ti_3_C_2_T_x_ MXenes [91]. Intriguingly, the magnetic properties of Ti_3_C_2_T_x_ MXene are quite dependent on doping it with different materials [92]. It has been found that iron doping in Ti_3_C_2_T_x_ can lead to strong ferromagnetic behavior [93]. The Fe atoms can also create localized magnetic moments due to their very nature, and facilitate interaction with the Ti atoms, to produce an increased overall magnetic moment on the Ti_3_C_2_T_x_ MXene. Furthermore, the magnetic properties of Ti_3_C_2_T_x_ MXene can also be improved by using cobalt (Co) and nickel (Ni) doping [94]. These transition metals have unpaired d electrons, which can contribute to the magnetic moment and allow Ti_3_C_2_T_x_ MXene to change from paramagnetic to ferromagnetic behavior [95]. Gadolinium (Gd) and neodymium (Nd) are also known rare earth elements, and their 4f electrons give them intense magnetic moments [96]. The magnetic properties of Ti_3_C_2_T_x_ MXene can be significantly enhanced by doping with rare earth elements, and remarkably strong ferromagnetism can be achieved at room temperature [97]. Fascinatingly, Ti_3_C_2_T_x_ MXene could support both the presence and absence of magnetism by virtue of boron doping [98]. Because boron atoms can produce local distortions on the lattice and change the spin distribution, they can increase the magnetic properties of Ti_3_C_2_T_x_ MXene. Meanwhile, the doping of nitrogen in Ti_3_C_2_T_x_ MXene has been demonstrated to enhance its magnetic behavior via interaction between the nitrogen atoms and titanium atoms in the Ti_3_C_2_T_x_ MXene structure [99]. These hybrid/composite/heterostructures of Ti_3_C_2_T_x_ MXene may also stimulate the modification of magnetic behavior. In addition, layered heterostructures of Ti_3_C_2_T_x_ MXene with other two-dimensional materials, such as graphene or TMDs, can also impact its magnetic properties [100]. The magnetic behavior of the heterostructures can be altered through interlayer interactions and charge transfer between layers. Consequently, we conclude that Ti_3_C_2_T_x_ MXene possesses interesting and tunable magnetic properties influenced primarily by surface terminations and doping with different materials [101].

**Figure 5:**
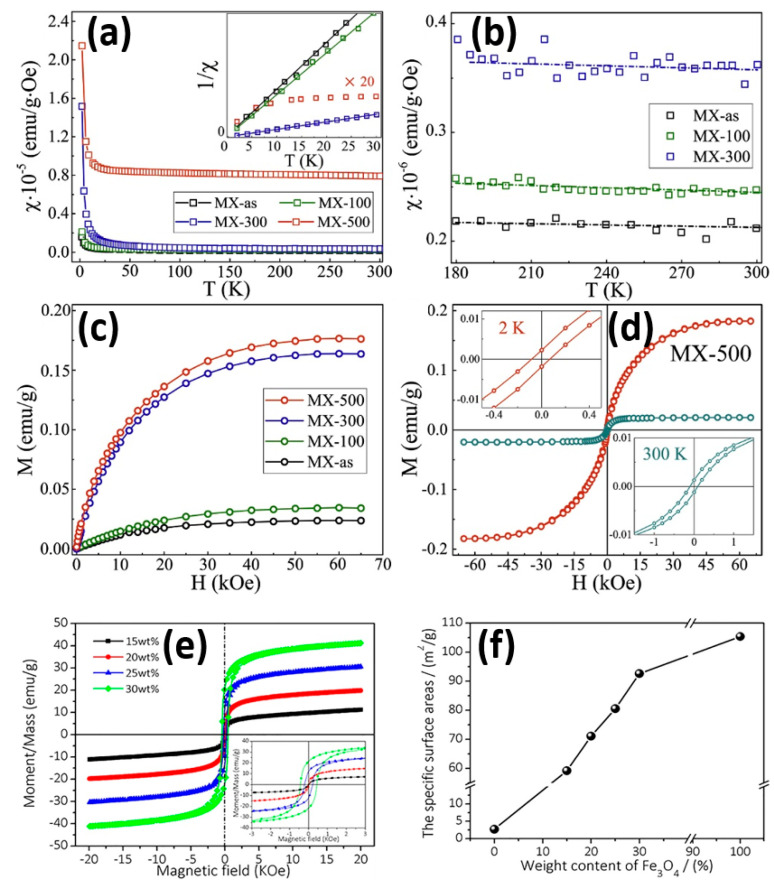
(**a**) Magnetic susceptibility, *χ*(*T*), curves measured from 2 to 3000 K under 1 kOe field; (**b**) closer examination of *χ*(*T*) curves for Ti_3_C_2_T_x_ (MX-as), Ti_3_C_2_T_x_ at annealing temperature 100 °C (MX-100), Ti_3_C_2_T_x_ at annealing temperature 300 °C (MX-300), and Ti_3_C_2_T_x_ at annealing temperature 500 °C (MX-500) in temperature range from 180 to 300 K; (**c**) magnetic moment (M) vs. applied field H plots at 2 K; and (**d**) hysteresis loop for MX-500 at 2 K and 300 K. Reproduced with permission from Ref. [102]. Copyright (2020) Elsevier. (**e**) Hysteresis loops of Fe_3_O_4_@Ti_3_C_2_T_x_ with different concentration of Fe_3_O_4_, and (**f**) specific surface area of Fe_3_O_4_, Ti_3_C_2_T_x_ MXene, and Fe_3_O_4_@Ti_3_C_2_T_x_ vs. Fe_3_O_4_ concentration plot. Reproduced with permission from Ref. [103]. Copyright (2019) Elsevier.

Zhang et al. discussed the magnetic properties of Ti_3_C_2_T_x_ (MX-as), Ti_3_C_2_T_x_ at an annealing of temperature 100 °C (MX-100), Ti_3_C_2_T_x_ at an annealing temperature of 300 °C (MX-300), and Ti_3_C_2_T_x_ at an annealing temperature of 500 °C (MX-500), as depicted in Figure 5a–d [102]. Figure 5a reveals the magnetic properties of monolayer Ti_3_C_2_T_x_ MXene samples before and after annealing in hydrogen (H_2_) at different temperatures, presenting the magnetic susceptibilities (*χ*) of the MX-as, MX-100, MX-300, and MX-500 samples as a function of temperature (2–300 K) in a 1 kOe field. It is observed that the MX-as, MX-100, and MX-300 samples show a combination of Curie and Pauli paramagnetism. However, MX-500 deviated severely from this behavior, signaling the onset of magnetic ordering. At low temperatures *χ*^−1^(*T*), MX-as, MX-100, and MX-300 could be well fitted by Curie’s law, but MX-500 deviates from Curie’s law, revealing ferromagnetic features appearing as temperature-dependent. Figure 5b divulges the magnified χ(T) curves of MX-as, MX-100, and MX-300 in the 180–300 K range, which show a dominance of Pauli paramagnetism. Figure 5c displays the magnetic moment (M) vs. the applied field (H) plot at 2 K under the applied field of 65 kOe. Successively, M_s_ increases progressively with annealing temperature, indicating substantial improvement in MX-500. Figure 5d shows the hysteresis loop of MX-500 at 2 K and 300 K. Ferromagnetic order was confirmed by the loop showing a clear coercive field (H_c_) of ~84 Oe and remnant magnetization (M_r_) ~0.002 emu/g at 2 K, which illustrates ferromagnetism. It is found that the magnetic properties are enhanced, including the emergence of ferromagnetism in MX-500, causing the formation of Ti-C vacancy pairs. Zhang et al. reported the enhanced microwave absorption properties of the Ti_3_C_2_T_x_, as revealed in Figure 5e,f [103]. As shown in Figure 5e, the Fe_3_O_4_@Ti_3_C_2_T_x_ composites exhibit ferromagnetic behavior with a clear magnetic hysteresis loop. It is found that the Fe_3_O_4_ content changes the values of key parameters, such as the saturation magnetization (M_s_), remnant magnetization (M_r_), and coercive field (H_c_). In particular, as the concentration of Fe_3_O_4_ grows, the saturation magnetization increases from 11.19 emu/g up to 41.20 emu/g. Therefore, these magnetic behaviors are attributed to microwave absorption performance factors, such as increased magnetic loss and energy dissipation. Figure 5f exhibits the evolution of the specific surface area at different contents of Fe_3_O_4_ in the Fe_3_O_4_@Ti_3_C_2_T_x_ composites. Interestingly, the presence of a 30 wt% of Fe_3_O_4_ in Fe_3_O_4_@Ti_3_C_2_T_x_ significantly increases its specific surface area to maximum as compared with Ti_3_C_2_T_x_. Furthermore, enhanced interface polarization is possible due to the improved specific surface area. However, it also increases the dielectric loss mechanisms, which are highly critical to successful microwave absorption. On the other hand, it is observed that the synergetic role of Fe_3_O_4_ in Fe_3_O_4_@Ti_3_C_2_T_x_ improves the magnetic and surface feature properties.

### 3.3. Electronic

Ti_3_C_2_T_x_ MXenes are most studied due to their fascinating electronic properties, where T stands for surface terminations, such as -OH, -O, and -F [53]. Ti_3_C_2_T_x_ MXene exhibits metallic or semi-metallic behavior with high electrical conductivity [104]. This is attributed to the presence of conductive Ti-C bonds within its layers and the contribution of the d electrons from titanium. In addition, the electronic band structure of Ti_3_C_2_T_x_ MXene can be tuned by modifying its surface terminations. Typically, Ti_3_C_2_T_x_ MXene with -OH and -O terminations exhibits metallic behavior, while -F terminations can open a small band gap, potentially altering its semiconducting properties [105]. The mobility of charge carriers in Ti_3_C_2_T_x_ MXene is relatively high, making it suitable for electronic device applications. Also, the surface terminations of the Ti_3_C_2_T_x_ MXene can influence the carrier concentration, density of states (DOS) near the Fermi level, and scattering mechanisms, influencing overall mobility [106]. Interestingly, it has been found that the presence of -O terminations tends to increase the DOS at the Fermi level, enhancing metallic properties; however, -F terminations can decrease the DOS at the Fermi level [107]. Also, it has been observed that the work function of Ti_3_C_2_T_x_ MXene is dependent on its surface chemistry and found to be between 4 and 5 eV, which is suitable for various electronic and optoelectronic applications [108]. Moreover, metal (Au, Pd, Pt, and Ag) doping can increase its inclusive electrical conductivity by introducing additional free electrons or holes in the Ti_3_C_2_T_x_ MXene [109]. In addition, doping with noble metals can also modify the work function, which makes Ti_3_C_2_T_x_ MXene more suitable for specific applications, such as catalysts or sensors [110]. Furthermore, non-metal dopants such as nitrogen, boron, and phosphorous can introduce states in the band gap or shift the conduction/valence bands, which effectively modulates the band gap of Ti_3_C_2_T_x_ MXene [111]. Excitingly, nitrogen doping is extremely important, as it changes metallic Ti_3_C_2_T_x_ MXene into semiconductor Ti_3_C_2_T_x_ MXene. It has also been studied that transition metals such as iron, cobalt, and nickel doping can introduce magnetic moments in Ti_3_C_2_T_x_ MXene, which makes it a more suitable candidate for spintronic applications [112]. Therefore, it is concluded that Ti_3_C_2_T_x_ MXene is a versatile material with tunable electronic properties, making it ideal for a wide range of applications. Further, doping with different materials allows for precise control over its electronic structure, conductivity, work function, and other vital properties, opening up new possibilities in various applications.

**Figure 6:**
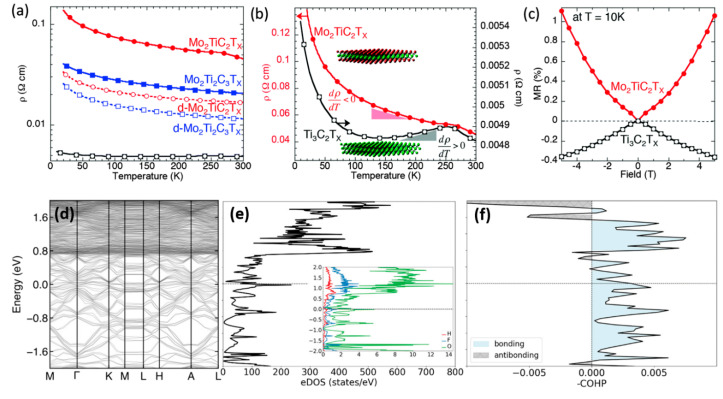
(**a**) The temperature-dependent resistivity ρ(T) plots of Mo_2_TiC_2_T_x_ (red), Mo_2_Ti_2_C_3_T_x_ (blue), and Ti_3_C_2_T_x_ (black) [delaminated films shown as dashed lines and pressed multilayered powders as solid lines]; (**b**) a comparison of the resistivity of Mo_2_TiC_2_T_x_ (red) and Ti_3_C_2_T_x_ (black), and the corresponding (**c**) measurements of the magnetoresistance as a function of the magnetic field at 10 K. Reproduced with permission from Ref. [113]. Copyright (2016) Royal Society of Chemistry. (**d**) The electronic band structure, (**e**) electronic density of states, and (**f**) -COHP of multilayer metallic Ti_3_C_2_T_x_ MXene. Reproduced with permission from Ref. [114]. Copyright (2024) Royal Society of Chemistry.

Anasori et al. reported the electronic properties of MXenes by modulating their transition metal layers, as exposed in Figure 6a–c [113]. Figure 6a illustrates the resistivity behavior of Ti_3_C_2_T_x_ MXene and Mo-containing MXenes (Mo_2_TiC_2_T_x_ and Mo_2_Ti_2_C_3_T_x_) as a function of temperature. Ti_3_C_2_T_x_ MXene presents metallic behavior with decreasing resistivity between 250 K and 130 K. On the other hand, Mo_2_TiC_2_T_x_ and Mo_2_Ti_2_C_3_T_x_ exhibit semiconductor-like behaviors, of which the resistivity increases with decreasing temperatures between 250 K and 130 K. Interestingly, it was found that replacing the outer titanium layers with molybdenum basically changed the electronic properties. Figure 6b demonstrates the resistivity differences between multilayered Mo_2_TiC_2_T_x_ and Ti_3_C_2_T_x_ MXenes. It shows the metallic behavior down to ~130 K of Ti_3_C_2_T_x_ MXene before an upsurge in its resistivity at lower temperatures. On the other hand, the resistivity of Mo_2_TiC_2_T_x_ increases monotonically with decreasing temperature and illustrates semiconducting behavior. Fascinatingly, it was concluded that the transition from metallic to semiconductor-like properties was due to molybdenum substitution. Figure 6c elucidates the differences in transport mechanisms in magnetoresistance measurements at 10 K. Negative MR is also found for the Ti_3_C_2_T_x_ MXene, ascribed to weak localization effects due to negative magnetoresistance. In contrast, the magnetoresistance shows positive behavior in the Mo_2_TiC_2_T_x_. Moreover, the magnetoresistance behaviors of Ti_3_C_2_T_x_ and Mo_2_TiC_2_T_x_ MXenes diverge from each other, suggesting fundamentally different electron transport mechanisms, solidifying molybdenum as a strong contributor to the electronic structure. Chu et al. described the electronic properties of Ti_3_C_2_T_x_ MXene by a first-principles study, as revealed in Figure 6d–f [114]. Figure 6d depicts the electronic band structure and bonding properties of multilayer Ti_3_C_2_T_x_ MXene. The electronic band structure and density of the states calculated from the multilayer MXene confirm metallic characteristics, as shown in Figure 6d,e. It is observed that the relation of surface terminations to the electronic properties of the multilayer Ti_3_C_2_T_x_ MXene structure is demonstrated by the presence of finite electronic states near the metal junction Fermi level, predominantly due to terminating groups H, F, and O. Figure 6f reveals a Crystal Orbital Hamilton Population (COHP) analysis of the bonding characteristics between adjacent layers. For H-O and H-F atomic pairs, the COHP analysis illustrates bonding features close to the Fermi level and finds the formation of H-bonds between adjacent terminating groups of adjacent layers of the Ti_3_C_2_T_x_ MXene. Fascinatingly, it is observed that this H-bonding between hydroxyl (OH) and F/O groups is vital for maintaining the structural integrity and stability of the multilayer Ti_3_C_2_T_x_ MXene and is a necessary factor in adhesion energy. Mainly, hydrogen bonds are formed between hydroxyl groups on one layer and fluorine or oxygen groups on neighboring layers of Ti_3_C_2_T_x_ MXene, leading to an additional increase in the interlayer adhesion energy and improving the stability of the multilayer structure.

Therefore, it is concluded from these optical, magnetic and electronic properties that MXenes could have a vital impact due to their gas sensing characteristics. Interestingly, the optical, magnetic, and electronic properties of Ti_3_C_2_T_x_ MXene are deeply interconnected, providing synergistic effects, which enhances its gas sensing capabilities. NO_2_ gas acts as an electron acceptor, reducing conductivity; however, reducing gases like NH_3_ increase it by donating electrons. Furthermore, the surface terminations (-OH, -O, -F) of Ti_3_C_2_T_x_ MXene significantly enhance its surface reactivity, providing active sites for gas adsorption. These terminations of Ti_3_C_2_T_x_ MXene modify the density of states near the Fermi level, influencing the electronic response and enabling a high sensitivity to adsorbed gases. In addition, the optical properties also contribute significantly to the gas sensing capabilities of Ti_3_C_2_T_x_ MXene. The material demonstrates surface plasmon resonance (SPR) in the visible and near-infrared regions, which can be modulated by changes in the local refractive index due to gas adsorption. This interaction causes shifts in the plasmonic response, enabling optical detection mechanisms. Fascinatingly, pristine Ti_3_C_2_T_x_ MXene is typically non-magnetic; however, defects, gas adsorption, and specific functional groups can induce its magnetic properties. Adsorption of certain gases, such as O_2_ and NO, can generate localized magnetic moments and alter Ti_3_C_2_T_x_ MXene’s magnetic behavior. This gas-induced magnetism provides an additional pathway for gas sensing, where changes in magnetic properties can be detected using magnetoresistance. Therefore, it is concluded that the optical, magnetic, and electronic properties of Ti_3_C_2_T_x_ MXene, combined with its 2D structure and tunable surface chemistry, make it a powerful material for gas sensing applications. Also, Ti_3_C_2_T_x_ MXene’s ability to detect gases through multiple mechanisms, such as changes in conductivity, optical shifts, and magnetic properties, offers a versatile platform for designing sensitive and selective sensors. Moreover, advancements in functionalization and defect engineering are expected to further enhance the potential of Ti_3_C_2_T_x_ MXene in gas sensing technologies.

## 4. Gas Sensing Study

Due to their comparatively high surface area, metallic conductivity, and hydrophilicity, 2D MXenes and their hybrids have demonstrated great promise for NH_3_ gas sensing applications, as discussed by Atkare et al. [115]. Their perspective only focuses on NH_3_ detection based on 2D MXenes and their hybrids. Due to the existence of different functional groups (-O, -OH, and -F) on the surface of the MXenes, the gas molecules could interact with the MXenes via different mechanisms, such as hydrogen bonding, van der Waals forces, and electrostatic interactions. Radhakrishnan et al. discussed the recent progress in 2D MXenes for NO_2_ gas sensing [116]. Their outlook only focuses on the NO_2_ gas sensing properties, mechanism, and the role of doping and heterostructures of 2D MXenes. Interestingly, the doping and heterostructures of 2D MXenes offer interactions that influence the electrical properties of the MXenes and can be used to detect various gases. In addition, Zhang et al. discussed MXene-based gas sensors for the monitoring of food safety [117]. Their perspective only emphasizes the detection of volatile organic compounds such as hydrogen sulfide (H_2_S), trimethylamine (TMA), acetone, and ethanol, which are critical for monitoring food quality and ensuring safety in agricultural products. These gases are typically released during the spoilage process, serving as key indicators of food degradation. Moreover, Ta et al. described a review of gas sensing and energy harvesting applications [118]. On the other hand, Yi et al. reported and focused only on the colorimetric detection of dipterex [119]. However, the present review focuses on the structural, morphological, optical, electronic, magnetic, and gas sensing properties of Ti_3_C_2_T_x_ MXene.

### 4.1. H_2_ Gas Sensing

The adsorption of hydrogen molecules on the surface of Ti_3_C_2_T_x_ MXene leads to a change in its resistance and is attributed to the high sensitivity of H_2_. Moreover, the response time of a Ti_3_C_2_T_x_ MXene sensor is required in practical applications. The fast adsorption–desorption dynamics of H_2_ are characteristic on the basis of the high conductivity of Ti_3_C_2_T_x_ MXene, leading to high response and recovery times. Additionally, the selectivity of Ti_3_C_2_T_x_ MXene is typically improved by surface functionalization or doping for H_2_ over other gasses [120]. Strong gas sensing selectivity performance can be achieved by doping various elements/compounds into the Ti_3_C_2_T_x_ MXene. In recent years, it was found that doping into Ti_3_C_2_T_x_ MXene can enhance its sensitivity, selectivity, and stability. In addition, Pd is well known as an excellent catalytic with a strong hydrogen adsorption capability. The dissociation of hydrogen molecules on Pd sites on Ti_3_C_2_T_x_ MXene improves its H_2_ sensitivity, fast response, and selectivity. Research by Nam et al. demonstrated that Pd-functionalized Ti_3_C_2_T_x_ MXene exhibited significantly improved H_2_ sensing performance, with lower detection limits, selectivity, and quicker response times [121]. On the other hand, Ti_3_C_2_T_x_ MXene@Pd has shown improved H_2_ sensing performance, as reported by Zhu et al., where the sensor exhibited higher flexibility, sensitivity, and faster recovery times compared to undoped Ti_3_C_2_T_x_ MXenes [122]. Qui et al. discussed a 3D PdCu/Ti_3_C_2_T_x_ MXene sensor, which demonstrated enhanced sensitivity and selectivity for H_2_ at room temperature [123]. Significant advancements towards H_2_ gas detection for energy systems and industrial safety are realized by the 3D PdCu-modified Ti_3_C_2_T_x_ MXene sensor (PdCu/Ti_3_C_2_T_x_). Interestingly, its unique architecture improves active site exposure and H_2_ gas diffusion to enable a fast 4 s response time, ultra-low detection limit (0.1% H_2_), and superior selectivity, stability, and repeatability. Moreover, by promoting synergistic interaction, the PdCu and Ti_3_C_2_T_x_ MXene heterostructure improves performance.

**Figure 7:**
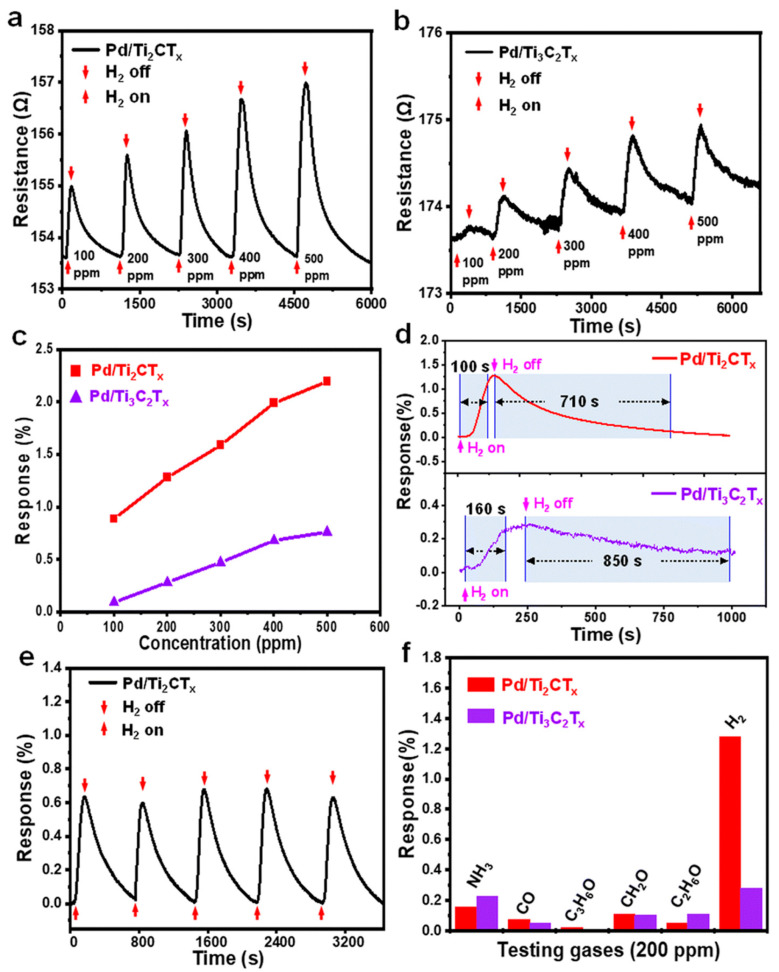
(**a**) Dynamic response–recovery curves of Pd/Ti_2_CT_x_ and (**b**) Pd/Ti_3_C_2_T_x_ sensors to varying concentrations of H_2_ at room temperature. (**c**) Correlation between H_2_ concentration and response for Pd/Ti_2_CT_x_ and Pd/Ti_3_C_2_T_x_ sensors. (**d**) Extracted response and recovery times from the response–recovery curves of Pd/Ti_2_CT_x_ and Pd/Ti_3_C_2_T_x_ sensors at 200 ppm of H_2_. (**e**) Cycling response–recovery curves of Pd/Ti_2_CT_x_ sensor at 200 ppm of H_2_. (**f**) Selectivity test results of Pd/Ti_2_CT_x_ and Pd/Ti_3_C_2_T_x_ sensors. Reproduced with permission from Ref. [124]. Copyright (2023) Royal Society of Chemistry.

Yang et al. discussed the H_2_ gas sensing performance of Pd/Ti_3_C_2_T_x_ sensors, as shown in Figure 7a–f [124]. Figure 7a,b show dynamic response–recovery curves, which indicate that the Pd/Ti_3_C_2_T_x_ sensor shows reversible behavior when exposed to H_2_. The Pd/Ti_3_C_2_T_x_ sensors exhibit p-type semiconductor-like behavior when exposed to H_2_ gas and have increased sensor resistance. After H_2_ removal, the Pd/Ti_3_C_2_T_x_ sensor heals to its baseline resistance, showing effective desorption and reversibility. Figure 7c quantifies the relationship between H_2_ (100–500 ppm) concentration and the responses of the Pd/Ti_2_CT_x_ and Pd/Ti_3_C_2_T_x_ sensors. It is observed that there is nearly three times as much of a response, at 2.2%, to H_2_ at 500 ppm for Pd/Ti_2_CT_x_ compared to Pd/Ti_3_C_2_T_x_, which had a response of 0.76%. Figure 7d displays the response and recovery times of the Pd/Ti_2_CT_x_ and Pd/Ti_3_C_2_T_x_ sensors at 200 ppm H_2_. Fascinatingly, the Pd/Ti_2_CT_x_ sensor illustrates significantly faster kinetics (100 s response and 710s recovery time) than the Pd/Ti_3_C_2_T_x_ sensor (160 s response and 850 s recovery time). These superior interaction dynamics of the Pd/Ti_2_CT_x_ heterointerface are also characteristic of a rapid response and recovery, marking the faster adsorption and desorption of hydrogen gas molecules. Figure 7e demonstrates the stability and reproducibility of the Pd/Ti_2_CT_x_ sensor through repeated response–recovery cycles at 200 ppm H_2_. These robust and reliable results of consistent Pd/Ti_2_CT_x_ sensor responses over multiple cycles confirm its robustness and reliability for practical applications. In Figure 7f, the selectivity of the Pd/Ti_2_CT_x_ and Pd/Ti_3_C_2_T_x_ sensors is evaluated by comparing their responses to NH_3_, CO, C_3_H_6_O, HCHO, C_2_H_5_OH, and H_2_. The Pd/Ti_2_CT_x_ exhibits the highest response to H_2_ and thus demonstrates excellent selectivity for targeted gas detection applications. Therefore, it was concluded that the thinner structure of the Ti_2_CT_x_ sensor is responsible for its superb H_2_ gas sensing properties, which result from an increase in the specific surface area, more active sites for adsorption, and a more effective charge transfer at the Pd–MXene heterointerface.

### 4.2. NH_3_ Gas Sensing

The development of a proton exchange membrane fuel cell (PEMFC) sensor based on Pt/Ti_3_C_2_T_x_ MXene for the detection of ammonia (NH_3_) has been studied [125]. Pt nanoparticles supported on Pt/Ti_3_C_2_T_x_ MXene nanosheets show enhanced sensitivity (456 nA/ppm) over conventional Pt/C sensors (313 nA/ppm) at room temperature with a limit of detection (LoD) of as low as 15 ppb. This remarkable performance arises from two synergistic effects: (i) the intrinsic high corrosion resistance and electrical conductivity of Ti_3_C_2_T_x_ ensures stable and efficient charge transfer, and (ii) the enhanced anchoring effect between Pt particles and the Ti_3_C_2_T_x_ nanosheet reduces catalyst detachment and improves stability. As a result of these characteristics, the sensor provides exceptional selectivity, reproducibility, and long-term stability. Moreover, the fabrication of a SnO_2_/Ti_3_C_2_T_x_ heterojunction sensor for the sensing of ammonia (NH_3_) gas has been studied [126]. The study develops SnO_2_/Ti_3_C_2_T_x_ heterojunction nanocomposites for highly efficient and reliable NH_3_ detection at room temperature. It is observed the SnO_2_/Ti_3_C_2_T_x_ heterojunction sensor elucidates a higher response than the Ti_3_C_2_T_x_ sensor due to the synergistic effects of the heterojunction interface, which promotes efficient charge transfer and gas adsorption. Moreover, the SnO_2_/Ti_3_C_2_T_x_ heterojunction sensor’s high sensitivity is further evidenced by the detection of NH_3_ at the ppb level, which makes it an attractive candidate for environmental monitoring and industrial safety. Fascinatingly, by combining the gas sensing properties of SnO_2_ with the surface porosity and the few lamellar structures of Ti_3_C_2_T_x_, this combination is uniquely sensitive to NH_3_ gas molecule capture and release. Most interestingly, the SnO_2_/Ti_3_C_2_T_x_ heterojunction sensor runs at room temperature. Therefore, it does not consume additional external heating which greatly contributes to lower energy expenditure. The combination of these features fits well with the worldwide movement towards energy-efficient technologies. The SnO_2_/Ti_3_C_2_T_x_ heterojunction sensor shows high sensitivity, fast response and recovery times, and excellent selectivity and stability. In addition, the SnO_2_/Ti_3_C_2_T_x_ heterojunction sensor’s ability to select NH_3_ from other gases addresses a common problem with gas sensing technologies. Interestingly, the GaN/Ti_3_C_2_T_x_ composite was synthesized via hydrothermal and nitridation processes, integrating the high conductivity of Ti_3_C_2_T_x_ MXene with the excellent sensing properties of GaN nanorods [127]. This innovative combination achieves improved NH_3_ sensing performance at room temperature, solving the high-temperature limitation of GaN nanorods. At room temperature, the GaN/Ti_3_C_2_T_x_ composite sensor demonstrates a lower detection limit of 0.5 ppm and a response 3.4 times higher than Ti_3_C_2_T_x_ alone. However, this study does not address the cost and scalability issues of mass production. In addition, work at extreme conditions, including RH > 80% or varying temperatures, is unexplored. The gas sensing mechanism for other gases is also not fully discussed. Stimulatingly, the use of self-assembled Ti_3_C_2_T_x_ MXene thin films as high-performance NH_3_ sensors has been explored [128]. In this study, the development of a high-performance NH_3_ gas sensor using Ti_3_C_2_T_x_ MXene nanosheet thin films grown by enhanced interfacial self-assembly is investigated. Ultra-thin films (<10 nm thick) with excellent sensitivity (1.92% at 1 ppm NH_3_), a response time of 179 s, and a recovery time of 612 s at room temperature result from this. Despite the occurrence of a variety of chemical changes related to humidity variation (10% to 90% RH), the Ti_3_C_2_T_x_ MXene thin-film sensor maintained remarkably stable performance and reproducible measures throughout wide humidity levels, suitable for environmental monitoring and other practical applications. The Ti_3_C_2_T_x_ MXene thin-film sensor shows superior sensitivity to low concentrations of ammonia (1 ppm) and rapid response and recovery times. It works without requiring a high-temperature heating element and reduces energy use at room temperature. Such a fabrication process is scalable and cost-effective, potentially resulting in large-scale productions. Remarkably, one study discusses the use of α-Fe_2_O_3_/Ti_3_C_2_T_x_ MXene nanocomposites to detect NH_3_ at room temperatures in a high-performance NH_3_ sensor [129]. Because of their hierarchical nanostructures achieved by a single hydrothermal method, these α-Fe_2_O_3_/Ti_3_C_2_T_x_ MXene composites have high sensitivity to NH_3_ (18.3% for 5 ppm at room temperature), fast response and recovery (<2.5 s), good reversibility, and good stability. The plausible NH_3_ sensing mechanism is supported by density functional theory (DFT) calculations that reveal a strong adsorption capacity and high gas selectivity. The results present the unique hierarchical structure of α-Fe_2_O_3_ nanoflower features and Ti_3_C_2_T_x_ MXene sheets’ hybrid heterojunctions that improve NH_3_ gas sensing properties at room temperature. More investigation is needed to determine the stability and compatibility of the α-Fe_2_O_3_/Ti_3_C_2_T_x_ MXene composite in practical applications, such as wearable devices, and under variable environmental conditions.

**Figure 8:**
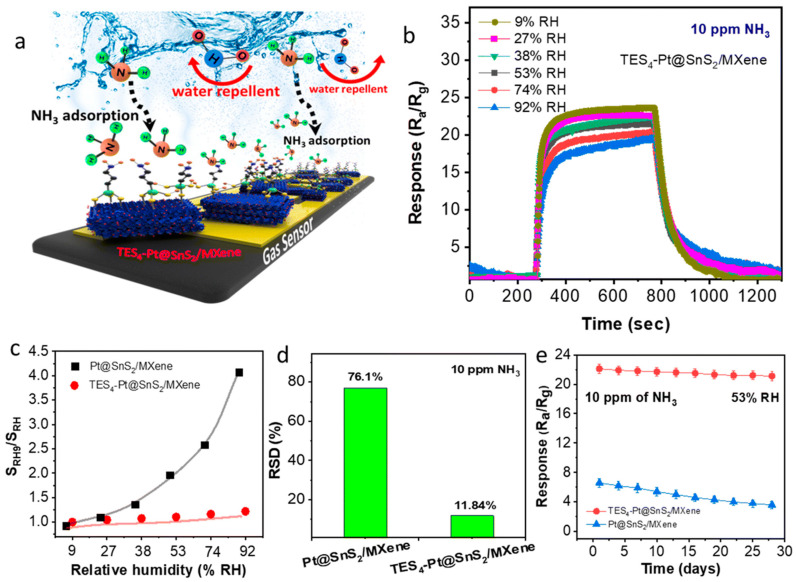
(**a**) A clear and detailed diagram of the gas sensing mechanism of the TES_4_-Pt@SnS_2_/Ti_3_C_2_T_x_ MXene sensor in the existence of humidity. (**b**) The response curves of the TES_4_-Pt@SnS_2_/Ti_3_C_2_T_x_ MXene sensor to 10 ppm NH_3_ under varying RHs. (**c**) The variation of S_RHO_/S_RH_ with relative humidity. (**d**) The RSDs (%) of the Pt@SnS_2_/Ti_3_C_2_T_x_ MXene and TES_4_-Pt@SnS_2_/Ti_3_C_2_T_x_ MXene sensors. (**e**) The stability of the Pt@SnS_2_/Ti_3_C_2_T_x_ MXene and TES_4_-Pt@SnS_2_/Ti_3_C_2_T_x_ MXene sensors over 30 days at 10 ppm of NH_3_. Reproduced with permission from Ref. [130]. Copyright (2024) Royal Society of Chemistry.

Ranjith et al. discussed the NH_3_ gas sensing performance of a Pt@SnS_2_/Ti_3_C_2_T_x_ MXene sensor, as revealed in Figure 8a–e [130]. Figure 8a shows a schematic representation of the gas sensing mechanism of the Pt@SnS_2_/Ti_3_C_2_T_x_ MXene sensor under the presence of humidity. By avoiding the adverse effects of water molecules on the sensing surface, the TES-functionalized Pt@SnS_2_/Ti_3_C_2_T_x_ MXene sensor displays a strategic design. TES functionalization, which gives it a hydrophobic nature, is such that there is no or very little interaction of water with the TES-functionalized Pt@SnS_2_/Ti_3_C_2_T_x_ MXene sensor’s active surface. Therefore, it is concluded that the TES-functionalized Pt@SnS_2_/Ti_3_C_2_T_x_ MXene sensor has a sufficiently high sensitivity and reliability even in a highly humid environment, which is a challenging matter for conventional sensors. Figure 8b depicts the response curves of the TES_4_-Pt@SnS_2_/Ti_3_C_2_T_x_ MXene sensor towards 10 ppm of NH_3_ within a range of relative humidity (RH) levels (9%, 27%, 38%, 53%, 74%, and 92%). The TES_4_-Pt@SnS_2_/Ti_3_C_2_T_x_ MXene sensor reveals a reasonably retained response to a fairly wide humidity range. Figure 8c depicts the SRH_0_/SRH ratio vs. humidity plots of the TES_4_-Pt@SnS_2_/Ti_3_C_2_T_x_ MXene gas sensor. The TES-functionalized TES_4_-Pt@SnS_2_/Ti_3_C_2_T_x_ MXene sensor displays a stable SRH_0_/SRH ratio, confirming minimal degradation of performance with increasing RH. This stability demonstrates that TES functionalization can suppress humidity-induced interference. Figure 8d illustrates the RSD values of the TES_4_-Pt@SnS_2_/Ti_3_C_2_T_x_ MXene and Pt@SnS_2_/Ti_3_C_2_T_x_ MXene sensors. Its reliability and constant function under varying humidity conditions are better represented in the RSD values of the TES_4_-Pt@SnS_2_/Ti_3_C_2_T_x_ MXene sensor. Figure 8e shows the long-term stability of the TES_4_-Pt@SnS_2_/Ti_3_C_2_T_x_ MXene and Pt@SnS_2_/Ti_3_C_2_T_x_ MXene sensors. In long-term applications, the TES_4_-Pt@SnS_2_/Ti_3_C_2_T_x_ MXene sensor remains responsive with minimal loss. On the other hand, the Pt@SnS_2_/Ti_3_C_2_T_x_ MXene sensor with non-functionalized Pt shows more severe degradation over time. Therefore, it is concluded that the TES_4_-Pt@SnS_2_/Ti_3_C_2_T_x_ MXene exhibits excellent NH_3_ detection under a wide range of challenging humidity conditions. Interestingly, TES functionalization incorporation in the TES_4_-Pt@SnS_2_/Ti_3_C_2_T_x_ MXene sensor notably enhances humidity tolerance, stability, and overall sensing reliability, thereby becoming a very promising candidate for real-world applications.

### 4.3. VOCs Gas Sensing

The recent literature highlights the exceptional potential of Ti_3_C_2_T_x_ MXenes for VOC sensing. The metallic Ti_3_C_2_T_x_ MXene sensor discussed by Kim et al. showed enhanced sensitivity and selectivity for VOC sensing [93]. This work is significant as it addresses a critical challenge in solid-state gas sensing based on the Ti_3_C_2_T_x_ MXene sensor, providing ultra-high sensitivity for detecting volatile organic compounds (VOCs) at the parts-per-billion (ppb) level. The feasible applications of VOC sensing include environmental monitoring or the early detection of diseases, where trace-level sensitivity can be life-saving. The sensor operates with high metallic conductivity, low electrical noise, and a fully functionalized surface of Ti_3_C_2_T_x_ MXene for an unprecedented detection limit of 50–100 ppb for VOCs at room temperature. Furthermore, the Ti_3_C_2_T_x_ MXene sensor exceeds conventional semiconductor-based sensors regarding signal-to-noise ratio, with a result which is two orders of magnitude higher than in other 2D materials. The findings are critical to using functionalized metallic Ti_3_C_2_T_x_ MXene for ultra-sensitive sensing. Bhardwaj et al. discussed VOC detection based on a SrTiO_3_-passivated Ti_3_C_2_T_x_ MXene sensor [131]. In this study, a novel way to enhance the humidity tolerance of Ti_3_C_2_T_x_ MXene-based sensors for volatile organic compound (VOC) sensing is presented by passivating the hydrophilic Ti_3_C_2_T_x_ MXene surface with a superhydrophobic SrTiO_3_ (STO) overlayer. Interestingly, the sensing properties of Ti_3_C_2_T_x_ MXenes are combined with the moisture-blocking properties of STO, a known hydrophobic perovskite. From an application point of view, this approach reduces humidity interference, a significant problem in gas sensing applications. The SrTiO_3_-passivated Ti_3_C_2_T_x_ MXene sensor exhibits good selectivity, repeatability, and long-term stability, with a detection limit (LOD) down to 40 ppb. These features offer promise for real-time VOC detection using an SrTiO_3_-passivated Ti_3_C_2_T_x_ MXene sensor. Overall, this work provides a scientifically sound strategy to improve the humidity tolerance of SrTiO_3_-passivated Ti_3_C_2_T_x_ MXene-based VOC sensors. Tian et al. discussed the ethanol gas sensing properties of a carbon quantum dots (CQDs)-sensitized Ti_3_C_2_T_x_ MXene sensor [132]. Integrating Ti_3_C_2_T_x_ MXene and carbon quantum dots (CQDs) into a composite, which exploits the photoresponsive properties of CQDs and high conductivity of Ti_3_C_2_T_x_ MXene, is the novelty of the composite. Its improved specific surface area, charge transfer, and photoelectronic properties result in the increased detection of ethanol by using the CQDs/Ti_3_C_2_T_x_ MXene sensor. Also, the study signifies the roles of ultraviolet (UV) irradiation in improving CQDs/Ti_3_C_2_T_x_ MXene sensor efficiency by reducing recovery time. The practicality of the CQDs/Ti_3_C_2_T_x_ MXene sensor’s requirement of a specific operating temperature (140 °C) may increase energy consumption, making it less environmentally friendly. In addition, while UV irradiation will shorten recovery time, the dependence on UV light may restrict its utility in low light or off-grid conditions. Song et al. reported the HCHO and triethylamine gas sensing properties of a ZnO/Ti_3_C_2_T_x_ MXene heterostructure sensor [133]. A scientifically robust approach to developing formaldehyde and trimethylamine sensing performance utilizing a ZnO/Ti_3_C_2_T_x_ MXene heterostructure sensor is presented and shown to be successful in measuring the critical indicators of seafood freshness. The ZnO/Ti_3_C_2_T_x_ MXene heterostructure sensor’s versatility demonstrated in its response to formaldehyde at room temperature and triethylamine under thermal conditions is remarkable. However, some limitations exist, such as external light activation and temperature adjustment for specific gas detection, its performance at lower concentrations below 100 ppm or in real-world mixed gas detection, and the fact the thermal energy required for triethylamine sensing may impair its usefulness in energy-restricted environments. Song et al. reported n-butanol recognition using an Fe_2_O_3_/Ti_3_C_2_T_x_ MXene heterostructure sensor [134]. Interestingly, Schottky heterojunctions and synergistic effects were found in the gas sensing performance of the innovative n-type Fe_2_O_3_ nanoparticles/n-type Ti_3_C_2_T_x_ MXene sensor. The Fe_2_O_3_/Ti_3_C_2_T_x_ MXene heterostructure sensor performs better due to its high conductivity, allowing for the faster transfer of carriers and a reduction in resistance, and abundant surface defects that boost the adsorption and reaction of gas molecules. Furthermore, the study delivers a roadmap to constructing a gas-sensitive Fe_2_O_3_/Ti_3_C_2_T_x_ MXene heterostructure sensor to detect ppb-level gas. Nevertheless, its drawbacks may be synthesis difficulties in preparing Fe_2_O_3_/Ti_3_C_2_T_x_ MXene heterostructures on a large scale because of the possible degradation of Ti_3_C_2_T_x_ MXene under some environmental conditions.

**Figure 9:**
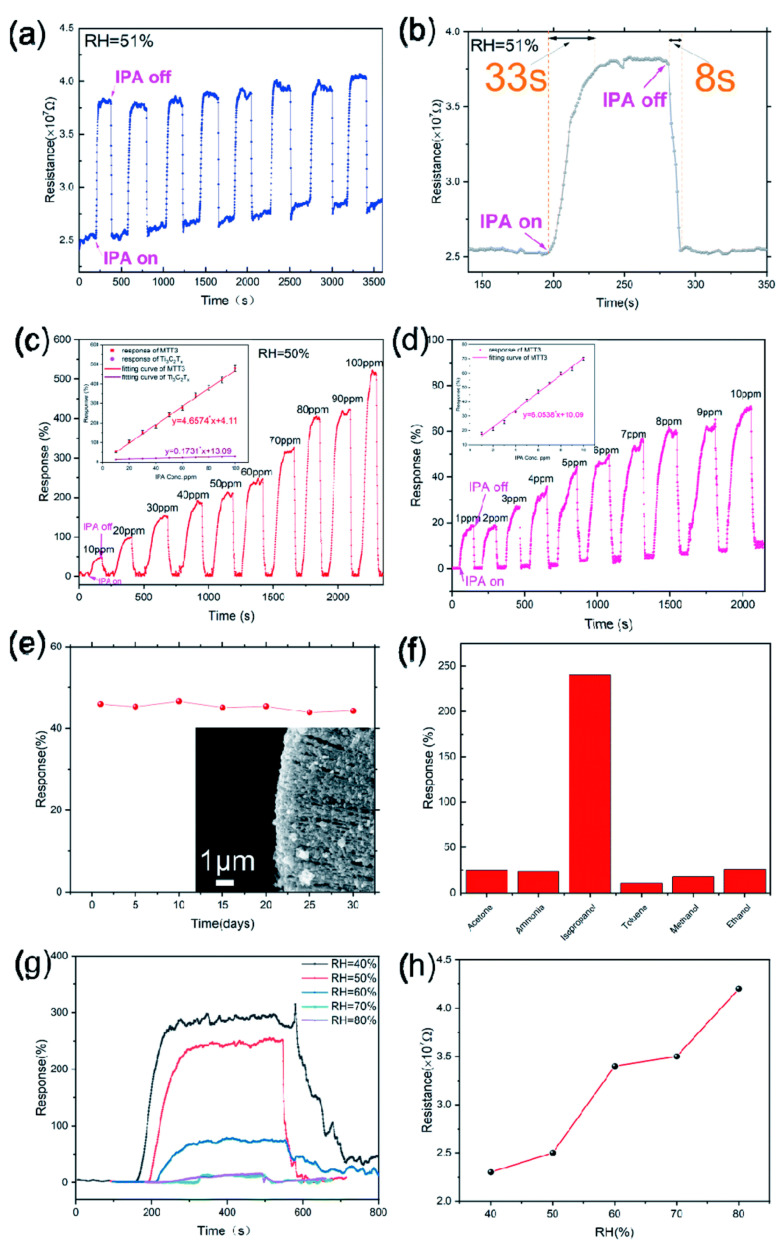
(**a**) The repeatability of the MoO_3_/TiO_2_/Ti_3_C_2_T_x_ ternary (MTT3) composite sensor for detecting 5 ppm isopropanol; (**b**) the response and recovery times of the MTT3 composite sensor for 5 ppm isopropanol; (**c**) the response–recovery curves of the MTT3 composite sensor for isopropanol concentrations of 10, 20, 40, and 100 ppm at 50% relative humidity; (**d**) the response–recovery curves of the MTT3 composite sensor for isopropanol at low concentrations ranging from 1 ppm to 10 ppm; (**e**) the long-term stability of the MTT3 composite sensor for measuring 5 ppm isopropanol for approximately 30 days; (**f**) the selectivity of the MTT3 composite sensor to various gases with a concentration of 50 ppm; (**g**) the dynamic response–recovery curves of the MTT3 composite sensor for isopropanol at concentrations of 50 ppm in relative humidity levels of 40 to 80%; and (**h**) the baseline resistance of the MTT3 composite sensor over a range of relative humidity levels 40–80%. Reproduced with permission from Ref. [135]. Copyright (2022) Royal Society of Chemistry.

Yao et al. reported isopropanol detection at room temperature using an MoO_3_/TiO_2_/Ti_3_C_2_T_x_ nanocomposite sensor, as depicted in Figure 9a–h [135]. Figure 9 also shows the comprehensive performance evaluation of the MoO_3_/TiO_2_/Ti_3_C_2_T_x_ (MTT3) composite-based gas sensor for isopropanol detection. Figure 9a illustrates multiple exposures of the sensor to 5 ppm isopropanol, during which it displays a stable response of approximately 45% in all cycles with repeatability. Figure 9b shows a remarkable response time of 33 s and a recovery time of 8 s for the MTT3 sensor for 5 ppm isopropanol. Figure 9c,d depict the dynamic response curves of the MTT3 sensor with varying isopropanol concentrations, revealing a linear relationship between the response and isopropanol concentration, confirming the MTT3 sensor’s sensitivity and precision. Interestingly, 50.45 ppb is the limit of detection (LOD), and 168.17 ppb is the limit of quantification (LOQ), demonstrating the MTT3 sensor’s ability to detect trace levels of isopropanol. Figure 9e exhibits the 50 ppm isopropanol testing results of the MTT3 sensor over 30 days, elucidating brilliant stability. Figure 9f further demonstrates the MTT3 sensor’s selectivity capability in distinguishing other potential interfering gases. Selectivity is a crucial characteristic because, in complex environments, multiple VOCs are present, which requires a suitable detection of desired gas. Figure 9g,h display that the MTT3 sensor’s performance degraded with varying humidity levels. However, above 80% RH, the MTT3 sensor’s response was reduced, possibly due to water vapor interference. Also, it was found that the MTT3 sensor’s performance returned to baseline after exposure to lower humidity, thereby showing the reversibility. Interestingly, the MTT3 sensor demonstrates high sensitivity, fast response and recovery, long-term stability, and good selectivity toward isopropanol detection at room temperature.

### 4.4. NO_2_ Sensing

Ti_3_C_2_T_x_ MXene has gained significant attention in the research community for its potential applications in gas sensing, particularly for nitrogen dioxide (NO_2_) detection. Gua et al. discussed NO_2_ sensing based on a Ti_3_C_2_T_x_ MXene/CuO nanocomposite sensor [136]. The advantages of the Ti_3_C_2_T_x_ MXene/CuO nanocomposite sensor are its enhanced sensitivity to NO_2_, significantly faster response–recovery dynamics, and long-term robust operation. In addition, the uniformity of the performance of the Ti_3_C_2_T_x_ MXene/CuO nanocomposite sensor has not been studied for gases other than NO_2_, which impedes its potential utility. However, the Ti_3_C_2_T_x_ MXene/CuO nanocomposite sensor still overcomes the challenges of room-temperature NO_2_ gas sensing technology. Shin et al. discussed NO_2_ detection based on a ZnO-Ti_3_C_2_T_x_ MXene nanocomposite sensor [137]. Under the influence of microwave irradiation for 1–8 min, the NO_2_ sensing performance of ZnO-Ti_3_C_2_T_x_ MXene nanocomposites with varying Ti_3_C_2_T_x_ MXene contents was studied. The superiority in NO_2_ response over some conventional materials was attributed to the creation of ZnO-Ti_3_C_2_T_x_ MXene Schottky barriers, microwave-induced oxygen vacancies, a large surface area, and functional surface groups on Ti_3_C_2_T_x_ MXene. In addition, the sensor exhibited high reproducibility and repeatability over a six-month period, which indicates great potential for practical application. Furthermore, Ti_3_C_2_T_x_ MXene-functionalized materials may be degraded in prolonged exposure to high temperatures; however, this study shows that microwave-irradiated ZnO-Ti_3_C_2_T_x_ MXene nanocomposites hold the potential to be cost-effective and stable NO_2_ sensing applications. Tang et al. discussed NO_2_ sensing based on an M-Ti_3_C_2_T_x_ MXene sensor [138]. Using a molten salt immersion method, this study successfully modulates the intrinsic functional groups of Ti_3_C_2_T_x_ MXene. To overcome the high room-temperature NO_2_ detection limit, the sensor is introduced to tunable oxygen-functionalized surfaces. These modifications include O addition to surface titanium atoms and O substitution on edge carbon atoms to maximize -O functional groups and enhance gas sensing performance. DFT calculations verify the covalent bond formation between the -O group and NO_2_ gas molecules, enhancing adsorption capacity. Hu et al. discussed NO_2_ sensing based on a Ti_3_C_2_T_x_/TiO_2_/Au heterostructure sensor [139]. Here, a visible light-assisted gas sensor based on Ti_3_C_2_T_x_/TiO_2_/Au heterostructures for room-temperature detection with an extremely low detection limit of NO_2_ was studied. The Ti_3_C_2_T_x_/TiO_2_/Au heterostructure sensor displays superior performance, with sensitivity without light illumination. The Ti_3_C_2_T_x_/TiO_2_/Au heterostructure sensor unveils the theoretical and experimental investigations of localized surface plasmon resonance (LSPR) and localized photothermal effects to increase photon utilization and NO_2_ gas-sensitive reactions. Interestingly, Au nanoparticles are incorporated to enhance the sensitivity and selectivity of NO_2_ detection. Quick response and recovery times make the Ti_3_C_2_T_x_/TiO_2_/Au heterostructure sensor more suitable for real-time monitoring, a key requirement for Internet of Things (IoT) applications. The cost-effective, large-scale production of Ti_3_C_2_T_x_/TiO_2_/Au heterostructure sensors may be complicated by their complex fabrication process. In addition, this work demonstrates the potential of plasmonic photothermal sensitization to move gas sensor technologies toward their IoT applications.

**Figure 10:**
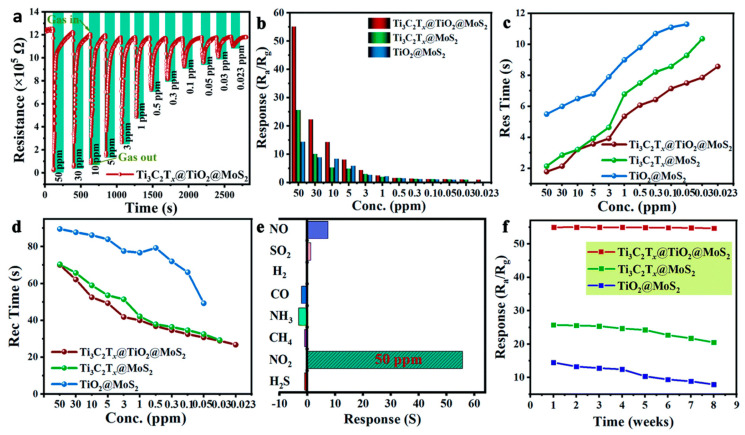
(**a**) Transient response curves of Ti_3_C_2_T_x_@TiO_2_@MoS_2_ sensor to NO_2_ at room temperature (RH = 23.4%). (**b**) Variation in response with concentration plot of TiO_2_@MoS_2_, Ti_3_C_2_T_x_@MoS_2_, and Ti_3_C_2_T_x_@TiO_2_@MoS_2_ sensors. (**c**,**d**) Response time vs. concentration plots of TiO_2_@MoS_2_, Ti_3_C_2_T_x_@MoS_2_, and Ti_3_C_2_T_x_@TiO_2_@MoS_2_ sensors. (**e**) Selectivity performance of Ti_3_C_2_T_x_@TiO_2_@MoS_2_ sensor. (**f**) Stability of TiO_2_@MoS_2_, Ti_3_C_2_T_x_@MoS_2_, and Ti_3_C_2_T_x_@TiO_2_@MoS_2_ sensors at 50 ppm NO_2_. Reproduced with permission from Ref. [140]. Copyright (2022) Royal Society of Chemistry.

Liu et al. reported NO_2_ gas sensing properties at room temperature using a Ti_3_C_2_T_x_@TiO_2_@MoS_2_ heterostructure sensor, as described in Figure 10a–f [140]. As shown in Figure 10, the Ti_3_C_2_T_x_@TiO_2_@MoS_2_ heterostructure sensor performs exceptionally well at NO_2_ detection at ambient temperatures. Figure 10a is the dynamic response of the Ti_3_C_2_T_x_@TiO_2_@MoS_2_ heterostructure sensor to varying concentrations of NO_2_. When exposed to NO_2_ gas, the Ti_3_C_2_T_x_@TiO_2_@MoS_2_ heterostructure sensor exhibits a rapid decrease in resistance, a characteristic behavior of a p-type semiconductor. To explore the Ti_3_C_2_T_x_@TiO_2_@MoS_2_ heterostructure sensor’s sensitivity to NO_2_, Figure 10b displays the magnitude of responses of different sensors (TiO_2_@MoS_2_, Ti_3_C_2_T_x_@MoS_2_, and Ti_3_C_2_T_x_@TiO_2_@MoS_2_) to 50 ppm of NO_2_. Figure 10c,d show the Ti_3_C_2_T_x_@TiO_2_@MoS_2_ heterostructure sensor’s speed in detecting and recovering from NO_2_ exposure. In addition, the Ti_3_C_2_T_x_@TiO_2_@MoS_2_ heterostructure sensor demonstrates a significantly higher NO_2_ sensing performance, which is attributed to the high conductivity of Ti_3_C_2_T_x_ but also to the intimate contact at the heterointerfaces allowing for fast electron transfer and efficient gas adsorption–desorption kinetics. As illustrated in Figure 10e, the selectivity of the Ti_3_C_2_T_x_@TiO_2_@MoS_2_ heterostructure sensor to NO_2_ over other gases, including H_2_, CO, CH_4_, NH_3_, and H_2_S, is remarkable. Figure 10f depicts the stability of the TiO_2_@MoS_2_, Ti_3_C_2_T_x_@MoS_2_, and Ti_3_C_2_T_x_@TiO_2_@MoS_2_ heterostructure sensors. It is observed that the sensors illustrate stability for up to eight weeks and five repeated tests for 50 ppm NO_2_. This stability reflects the stiffness of the electronic properties and the sensor’s structure, which indicates good long-term performance.

### 4.5. Humidity Sensing

Liu et al. propose a novel optical fiber relative humidity (RH) sensor using a tapered no-core fiber (TNCF) coated with Ti_3_C_2_T_x_ MXene [141]. The Ti_3_C_2_T_x_ MXene’s refractive index (RI) changes upon RH variations, allowing it to sense humidity by shifting the transmission spectrum. The advantages of the Ti_3_C_2_T_x_ MXene/TNCF sensor include a simple design, low cost, high reversibility, and excellent stability. A novel approach to optical fiber-based humidity sensing is demonstrated by the proposed sensor, exhibiting high sensitivity and fast response. Waheed et al. propose a novel 2D Ti_3_C_2_T_x_ MXene nanosheet and graphene oxide sensor for humidity sensing [142]. A novel, high-performance, and cost-effective humidity sensor was developed using 2D Ti_3_C_2_T_x_ MXene nanosheets as electrodes and graphene oxide (GO) as the sensing layer. The sensor device demonstrates rapid response and recovery times of 0.8 s and 0.9 s, respectively, while maintaining stability over 24 h. Density functional theory (DFT) simulations provided atomic-level insights into the humidity sensing mechanism, highlighting the formation of physical bonds between water molecules’ hydrogen atoms and the oxygen atoms in the OH groups of the GO. The findings underscore the potential of 2D material-based sensors for low-cost, flexible, and highly sensitive humidity detection. This study contributes to advancing 2D materials in sensor technology, offering promising implications for diverse humidity sensing applications. In addition, Yu et al. propose a novel Ti_3_C_2_T_x_ MXenes–RGO sensor for humidity sensing [143]. Based on the developed Ti_3_C_2_T_x_ MXenes–RGO sensor, the prospects for achieving a highly sensitive and accurate biosensor for respiratory moisture diagnostics in real-time have been successful. This idea could dramatically change the axis of health diagnostics, where respiratory patterns could be scanned without physical contact, and it could further enhance the sphere of environmental monitoring. Based on the results presented in the study, the Ti_3_C_2_T_x_ MXenes–RGO sensor could point in the direction of new techniques with higher non-invasiveness in healthcare and environmental science. In addition, Tang et al. propose a novel C_60_-OH/Ti_3_C_2_T_X_ nanocomposite sensor for humidity sensing [144]. Incorporating C_60_-OH with Ti_3_C_2_T_X_ nanostructures in high-frequency quartz crystal microbalance (QCM) sensors may enhance their moisture adsorption capacity and sensitivity, as well as response and recovery rates. This approach can benefit the humidity sensing profiles that need greater sensitivity and time responsiveness, like human noninteraction.

**Figure 11:**
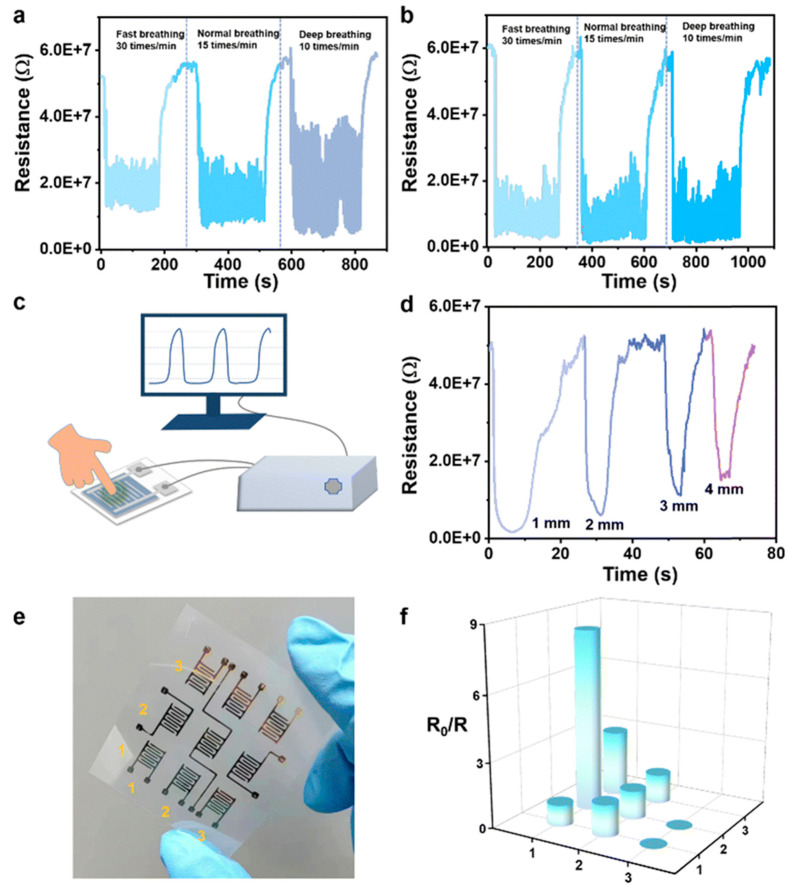
The Ti_3_C_2_T_x_/SnO_2_ sensor exhibits characteristic resistance responses to human breathing through the (**a**) nose and (**b**) mouth. (**c**) The graphic scheme of non-contact gesture monitoring. (**d**) The real-time resistance varies as a function of distance of 1–4 mm between a finger and the Ti_3_C_2_T_x_/SnO_2_ sensor. (**e**) A photograph of the printed Ti_3_C_2_T_x_/SnO_2_ sensor array shows its compact and potentially scalable design. (**f**) Testing of the Ti_3_C_2_T_x_/SnO_2_ sensor array is conducted, and the results confirm its practicality. Reproduced with permission from Ref. [145]. Copyright (2024) Royal Society of Chemistry.

Han et al. propose a novel Ti_3_C_2_T_x_ MXene/SnO_2_ heterostructure sensor for humidity sensing, depicted in Figure 11a–f [145]. The real-time resistance response of the Ti_3_C_2_T_x_ MXene/SnO_2_ heterostructure sensor during respiration in different rhythms and techniques is shown in Figure 11a,b in fast (30 bpm), normal (15 bpm), and slow (10 bpm) respiration. The study evidences the capability of the Ti_3_C_2_T_x_ MXene/SnO_2_ heterostructure sensor to distinguish between mouth and nose breathing. The research established that mouth breathing produces a lower resistance than nasal breathing, a factor attributed to the high humidity of the air leaving the mouth. These outcomes suggest the sensor’s capability for continuous respiratory monitoring, which would be very helpful when diagnosing sleep and other respiratory disorders. Figure 11c shows the schematic depiction of the contactless gesture monitoring process of the Ti_3_C_2_T_x_ MXene/SnO_2_ heterostructure sensor. Figure 11d displays the transient resistance characteristics of the Ti_3_C_2_T_x_ MXene/SnO_2_ heterostructure sensor. Interestingly, if a finger is moved closer to the sensor, the resistance changes, going to a higher resistance level if it moves closer to the sensor within a limit of 1–4 mm. This change in resistance is due to a shift in humidity from the original humidity caused by the evaporation of sweat. This capability reveals that the sensor is capable of sensing the proximity of an object without actually touching it, creating an avenue for ingenious touchless interfaces as well as progressive human–machine interaction. Figure 11e portrays a photograph of a printed Ti_3_C_2_T_x_ MXene/SnO_2_ heterostructure sensor array to visualize the sensor. Figure 11f describes the response results of the printed Ti_3_C_2_T_x_ MXene/SnO_2_ heterostructure sensor array. The Ti_3_C_2_T_x_ MXene/SnO_2_ heterostructure sensor array can recognize localized changes in resistance when a finger is in proximity to some of these specific positions, as shown in Figure 11e. This capability demonstrates the potential uses of large-area humidity mapping in environmental sensing, gesture-based systems, and high-precision industrial control.

## 5. Conclusions

Ti_3_C_2_T_x_ MXene is a promising material for next-generation gas sensors, owing to its excellent intrinsic properties of high electrical conductivity, large specific surface area, and tunable surface chemistry. With these characteristics, Ti_3_C_2_T_x_ MXene is suitable for gas sensing with high gas selectivity and sensitivity. By synthesizing, functionalizing, and integrating Ti_3_C_2_T_x_ MXene into gas sensing devices, recent advances have shown the potential of this material to achieve both fast response times and further enhanced performance through composite device formation.

### 5.1. Current Limitations and Challenges

Although much progress has been made so far in designing and fabricating stable and reproducible Ti_3_C_2_T_x_ MXene-based gas sensors, there are still significant hurdles to overcome in order to ensure sensor stability and reproducibility for the long term. However, despite these issues, such materials have not been widely adopted due to such factors as oxidative degradation, sensitivity to humidity, and differences in material quality. Beyond that, there are critical bottlenecks regarding the scalability of production methods and seamless integration with existing technologies to one another and the rest of the world.

### 5.2. Future Directions for Research and Development

To overcome these limitations and enable the commercialization of Ti_3_C_2_T_x_ MXene gas sensors, the following strategies are proposed: (i) establish methods for synthesis that are both scalable and economical and also deposit consistent material quality and yield; (ii) optimize the etching process as well as exploring other routes to produce Ti_3_C_2_T_x_ MXene; (iii) explore new possibilities in functionalization technology like the use of the new dopants and surface modifiers to boost selectivity and sensitivity; (iv) uncover hybrid structures and composites based on Ti_3_C_2_T_x_ MXene paired with other functional materials, to extract sensing capabilities not available in these material systems alone; (v) design strategies that improve Ti_3_C_2_T_x_ MXene sensors’ resistance to oxidation, humidity, and other environmental factors which deteriorate sensor performance; (vi) along with that, implement advanced surface treatments or protective coatings to achieve complete operational stability of the Ti_3_C_2_T_x_ MXene-based sensors; (vii) extend the application scope of Ti_3_C_2_T_x_ MXene-based gas sensors to include biomedical diagnostics, food safety monitoring, and hazardous gas detection in industrial environments; (viii) investigate Ti_3_C_2_T_x_ MXene’s potential in specialized sensing scenarios, such as detecting biomarkers or volatile organic compounds; and (ix) further, deployment into real-world applications must be seamless and the emphasis should be on compatibility with existing technologies.

By addressing these future directions, the research community can unlock the full potential of Ti_3_C_2_T_x_ MXene, paving the way for innovative applications and the widespread adoption of Ti_3_C_2_T_x_ MXene-based gas sensors. The continued evolution of this field promises to contribute significantly to developments in sensing technology, environmental monitoring, and public health.
